# Nanostrategies synergize with locoregional interventional therapies for boosting antitumor immunity

**DOI:** 10.1016/j.bioactmat.2025.05.016

**Published:** 2025-05-31

**Authors:** Ting Luo, Kunpeng Ma, Yi Zhang, Qingwen Xue, Jie Yu, Xing-Jie Liang, Ping Liang

**Affiliations:** a Department of Interventional Ultrasound, Fifth Medical Center of Chinese People's Liberation Army General Hospital, Beijing, PR China; b Laboratory of Controllable Nanopharmaceuticals, Chinese Academy of Sciences (CAS) Key Laboratory for Biomedical Effects of Nanomaterials and Nanosafety, CAS Center for Excellence in Nanoscience, National Center for Nanoscience and Technology, Beijing, PR China; c Department of Interventional Radiology, First Medical Center of Chinese People's Liberation Army General Hospital, Beijing, PR China; d University of Chinese Academy of Sciences, Beijing, PR China

**Keywords:** Locoregional interventional therapy, Immunotherapy, Nanomedicine, Tumor microenvironment

## Abstract

Compared with traditional surgical resection, systemic chemotherapy, or radiotherapy, locoregional interventional therapies (LITs) possess their own advantages of minimally invasive procedure and immunomodulatory effects in cancer treatment. Local ablation and intravascular interventional therapy represent excellent LIT candidate to combine with immunotherapy. Diverse nanomaterials with excellent biocompatibility show promises in modulating antitumor immunity. In this review, we summarized several immune-LIT combinations, discussed the following immunomodulatory effects, and presented the novel nanostrategies for synergizing with the combination therapy. With continuous optimization, further promotion of clinical translation will ultimately benefit patients with personalized and tailored cancer strategy.

## Highlights

• We enumerate common LITs and summarize the combined immunotherapies in clinic.
• We discuss the immunomodulatory effect after LITs and advanced nanostrategies.

## Graphical abstract

**Figure:**
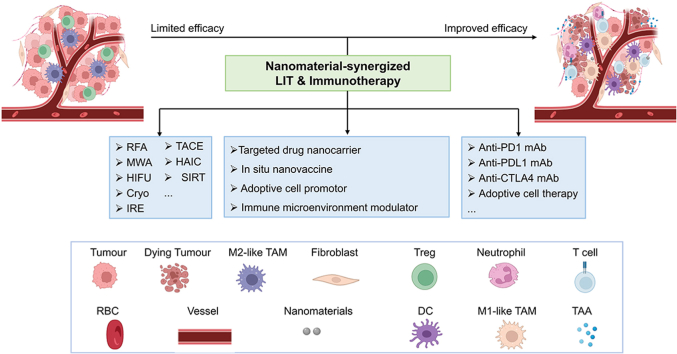

## 1 Introduction

Locoregional interventional therapy (LIT) refers to minimally invasive or even noninvasive medical procedures that use imaging guidance, such as X-ray, ultrasound, computer tomography (CT), or magnetic resonance imaging (MRI), to diagnose or treat various diseases, mainly in the cancer management [1,2]. These therapies often provide an alternative to traditional surgical resection, systemic chemotherapy, or radiotherapy, offering patients with shorter recovery time, less pain, and lower risks. LITs are generally categorized into the percutaneous ablative procedure and the transarterial embolization. The percutaneous approach mainly involves ablative therapies such as radiofrequency ablation (RFA), microwave ablation (MWA), cryoablation, laser ablation, and irreversible electroporation (IRE). High intensity focused ultrasound (HIFU) is a truly noninvasive ablative therapy with ultrasound irradiation. Intra-arterial treatments, pivotal in hypervascularized hepatocellular carcinoma (HCC) management, mainly include transarterial chemoembolization (TACE), hepatic arterial infusion chemotherapy (HAIC), and transarterial radioembolization or selective internal radiation therapy (SIRT). In addition to physical or chemical destruction on tumor, LITs also have substantial influence on tumor microenvironment (TME), including oxygen supply, cell debris release, and immune modulation [3].

With the rapid development in cancer immunotherapy, the integration of LITs is now under wide investigations, and owns the potential to revolutionize the treatment of solid tumors, which may significantly shift the future paradigm in clinic. Immune checkpoint inhibitors (ICIs) as well as cell-based immunotherapy represent prevalently clinically-used immunotherapies. Although satisfying results was shown in hematological malignancies (leukemia [4,5], multiple myeloma [[6], [7], [8]], lymphoma [[9], [10], [11]]), several challenges existing in solid tumors are insufficient expression of immune checkpoints, dense extracellular matrix, disabled antigen process and presentation, as well as suppressive immune components. Combined with LIT-induced immunomodulation, additional value may improve efficacy of current immunotherapies. However, the optimal timing for such combination therapy remains undetermined. And these combinations are lack of precise targeted strategy for single player or multiple immune interplay to trigger potent and prompt antitumor response.

Nanomedicine is an emerging therapeutic modality, which use nanosized biomaterial (1–1000 nm) for targeted drug delivery (small molecule agents, mRNA/siRNA, antibodies) to specific tissue, or the biomaterial itself act as a therapeutic role to overcome drug resistance and complement current clinical decision-making. Nanoparticles can be precisely engineered for size, surface charge, and ligand functionalization, allowing for improved targeting and controlled drug release. Owing to the diverse physicochemical properties of nanoparticles, less adverse events (AEs) and prolonged the circulation retention, nanomedicine is potentially improve the efficacy of cancer treatment and offer patients with advanced options. Unlike mesenchymal stem cell-derived exosomes [12] or radiotherapy-induced immunomodulation [13], which may have limitations in drug-loading capacity, potential immunogenicity, and transient immunostimulation, synthetic nanomaterials provide enhanced design flexibility, scalability, and reproducibility. Additionally, direct delivery through interventional techniques ensures high local drug concentrations, addressing systemic exposure challenges. Therefore, integrating nanomedicine into LIT offers significant advantages by enhancing both efficacy and safety.

**Fig. 1:**
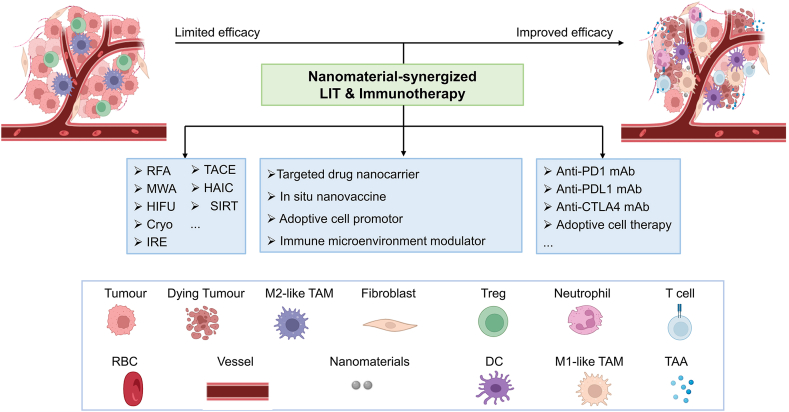
Schematic illustration of nanomaterial-synergized locoregional interventional therapies boosting immunotherapies.

In this review, we enumerate several common LITs in clinic and discuss their advantages and application. We also summarize the combined immunotherapies as the research outcomes from prospective clinical trials. We discuss the immunomodulatory effect after local cancer treatment and potential optimize direction. Accordingly, we outline some advanced preclinical nanostrategies to address the current challenges that hinder the wide application of immune-local interventional therapy combinations in cancer. We present our outlook on this emerging field at the interface of nanomaterial design and efficacy improvement, hoping nanomedicine opens a new avenue for enhanced LITs in future (Fig. 1).

## 2 Locoregional interventional therapy (LIT) and emerging cancer immunotherapy combinations

### 2.1 Ablation therapies

Local ablation is a minimally invasive procedure wherein high exogenous energy is introduced and harnessed to destroy malignant tissues, such as liver, kidney, lung, and bone tumors. In clinic, these imaging guided-ablative therapies, commonly under ultrasound, CT, or MRI modalities, can be useful alternatives to traditional surgical resection or organ transplantation in the context of small tumors. Additionally, for elderly patients or poor baseline status who cannot afford high risk, these microinvasive or even non-invasive treatment is of high significance.

RFA is currently the most widely applied, minimally invasive tumor ablation with low risk of AEs, which is a clinically reasonable option instead of surgery. RFA electrodes are inserted into targeted tumors under ultrasound guidance, followed by an 350–500 kHz alternating current generation.

MWA provides another ablative method causing coagulation necrosis of tumor. To deliver microwave energy, a thin needle-shaped antenna is inserted into target tissue under ultrasound guidance, followed by 915-2450 MHz frequencies of microwave intratumorally to cause immediate coagulation necrosis via high heating. When tumor is located close to important normal tissue and organs, sterile water or saline is injected around the ablation area, forming a water cushion that separates the targeted tissue from sensitive adjacent structures and absorb excessive ablative heat to provide protection. The ablation zone is spherical or ellipsoidal in shape which is critical to therapeutic efficacy in the light of heating-coverage area.

Laser ablation is microinvasive procedure which is percutaneous or via natural body openings, such as the mouth, nose, or urethra, to introduce focused laser at the target tissue energy by a thin fiber-optic probe. Laser light is absorbed by the tissue, converting it into heat, causing vaporize (for smaller, superficial tumors) or coagulate (for larger size) to destroy cancer.

HIFU is a truly non-invasive therapeutic technique that delivers high-frequency sound waves that converge at a focal point within the tissue under MRI or ultrasound guidance. The ultrasound waves create intense heat in a small, targeted area, which can destroy tissue, such as tumors, without affecting surrounding healthy tissue. At this focal point, the energy from the ultrasound waves causes rapid heating, leading to tissue coagulation or destruction. This is entirely non-invasive which is able to minimize damage to surrounding tissues and ensure patients to recovery within a short time after the procedure. HIFU is used for localized prostate, liver, pancreas, kidney, and breast cancer. In prostate cancer, it's an alternative to surgery or radiation for localized cases. However, HIFU is challenging in large, widespread, or multiple lesions. It may require multiple sessions, depending on the size and type of tissue being treated. HIFU continues to be studied and developed as a treatment option and shows great promise in wider applications.

Cryoablation is a minimally invasive procedure that destroys and freezes abnormal tissue, such as tumors. It involves inserting a cryoprobe, a thin, needle-like device, directly into the target tissue, guided by ultrasound, CT, or MRI. Cryoablation consists of repeated cooling-thawing cycles which involves an "ice ball' around the probe produced by liquid nitrogen or argon gas, and subsequent natural or heat-thawing, collectively inducing cell death. Cryoablation bring less uncomfortable experiences and less recovery time for patients. This technique is commonly used in treating certain cancers (such as prostate, kidney, liver, and lung cancers) and some benign tumors.

IRE is a minimally invasive technique used to destroy tumor cells by applying short, high-voltage electrical pulses. IRE works by disrupting the cell membranes through an electric field, causing irreversible damage to the cell structure and ultimately leading to cell death. Mechanically, several needle-like electrodes are inserted into and around the target tumor, and high-voltage pulses are then delivered to generate an intense electric field. Micropores are permanently produced in cell membranes, which is called electroporation, then cells undergo apoptosis (programmed death), and the immune system gradually clears the dead cells. With the non-thermal mechanism and tissue-preserving properties, IRE is much safer associated with a shorter recovery period and fewer side effects compared to traditional ablation methods. Ongoing research is promoting IRE applications, and it continues to show promises in treating challenging cancer.

Histotripsy, a brand-new non-invasive therapeutic ultrasound technology, uses high-amplitude focused ultrasound pulses to mechanically liquefy tissue into subcellular debris [14]. To be specific, histotripsy achieves tissue fractionation through the rapid formation, expansion, and collapse of microbubbles within the target tissue, effectively liquefying local tissue at a subcellular level without damaging surrounding structures. It surpasses traditional LITs due to the real-time monitoring, reduced heat sink effect, and most importantly, excellent immunostimulation. Histotripsy has been recently approved by the US Food and Drug Administration to treat liver tumors, with other clinical trials for benign prostatic hyperplasia [15] (NCT01896973) and calcified aortic stenosis (NCT03779620).

### 2.2 Embolization therapies

Embolization therapies are applied to treat hypervascularized tumors such as HCC, and renal carcinoma. TACE is a minimally invasive procedure primarily to treat liver cancer, especially in patients who cannot afford surgery [16,17]. Under X-ray guidance, TACE involves the selective delivery of chemotherapeutic drugs mixed with embolic agents directly into the tumor vessels. The blockage starves the tumor of oxygen and nutrients to limit growth, while the trapped chemotherapy remains concentrated within tumor. This allows precise target treatment with reduced systemic exposure and side effects. In HAIC, chemotherapy drugs are injected through hepatic artery via a catheter, allowing for a high concentration of chemotherapeutic drugs to reach the tumor site with reduced systemic side effects [[18], [19], [20]]. The catheter is often connected to an implanted infusion pump that can continuously or periodically offer the drug administration, allowing for steady and controlled chemotherapy. SIRT or radioembolization by intra-arterial injection of radioactive elements-loaded microspheres is increasingly applied in HCC [21,22], intrahepatic cholangiocarcinoma [23], and liver metastasis [24].

### 2.3 Immunotherapy combinations

Novel immunotherapies have markedly advanced the landscape of current cancer treatment, especially in the clinical setting of unresectable tumors. Clinically approved immunotherapies, such as ICIs and adoptive cell therapy, were gradually explored to synergize with interventional therapies. Emerging trials have investigated the safety and efficacy of combined therapies (Table 1). The indicators such as overall survival (OS), progression-free survival (PFS), overall response rate (ORR), and disease control rate (DCR) are commonly used to assess the efficacy of clinical trials, while treatment-related AEs are used to evaluate the safety.

**Table 1:** Clinical trials reporting interventional therapy combined with cancer immunotherapy.

Trial	Patient	Intervention	Efficacy	Safety
NCT03939975 [35]	Advanced HCC after sorafenib failure (n = 50)	At least one dose of nivolumab or pembrolizumab, 33 cases received subtotal RFA or MWA	ORR: 24 %; mTTP, mPFS, and mOS: 6.1 months, 5 months, and 16.9 months	Serious AEs in 14 % of patients
NCT04805736 [36]	Early-stage breast cancer (n = 60)	Single-dose camrelizumab alone (n = 20), MWA alone (n = 20), camrelizumab + MWA (n = 20)	57 patients received prescheduled surgery, and 3 women received neoadjuvant chemotherapy first	No grade 3–4 AEs
NCT01853618 [37]	Advanced HCC (n = 32)	Tremelimumab + subtotal RFA or chemoablation	Confirmed partial response rate: 26.3 %; 6 and 12-month probabilities of tumor PFS: 57.1 % and 33.1 %; mTTP: 7.4 months; mOS: 12.3 months	Grade 3–4 AEs in 53 % of patients
NCT01853618 [38]	Advanced biliary tract cancer (n = 20)	Tremelimumab + subtotal MWA	Confirmed partial response rate: 12.5 %; mPFS: 3.4 months; mOS: 6.0 months	Grade 3–4 AEs on 19 patients
NCT02626130 [39]	Metastatic renal cell carcinoma (n = 29)	Tremelimumab with (n = 15) or without (n = 14) cryoablation	ORR: 3.4 % in the combination therapy arm; mPFS: 3.3 months for all treated patients	Grade ≥3 AEs in 55 % of patients
NCT04102098 [25]	Patients with a high risk for HCC recurrence following resection or local ablation (n = 668)	Atezolizumab + bevacizumab every 3 weeks or active surveillance	mRFS: not reach	Atezolizumab + bevacizumab: 41 % (grade 3–4 AEs), 2 % (grade 5 AEs); active surveillance: 13 % (grade 3–4), <1 % (grade 5)
NCT02821754 [40]	Advanced BTC (intra- or extrahepatic cholangiocarcinoma, gallbladder cancer, or ampullary cancer) (n = 22)	Durvalumab and tremelimumab (Arm A, n = 10), combination of tremelimumab and durvalumab with RFA (Arm B, n = 12)	mPFS: 3.3 months in Arm A, 2 months in Arm B; mOS: 6.1 months in Arm A, 5.7 months in Arm B	Hematologic-related grade 3–4 AEs in 19 patients and 22 of non-hematologic AEs in 22 patients
NCT02250014 [41]	Low to intermediate-risk localized prostate cancer (n = 20)	Cryoablation alone (Control group, n = 10) or + GM-CSF (Treatment group, n = 10)	At 4 weeks: Treatment group shows an increase of 2.8 % in cancer antigen-related antibodies; Control group shows a decrease of 18 %. At 12 weeks: the treatment group shows an increase of 25 %, Control group shows a decrease of 9 %	No grade 3 AEs
KCT0000008 [27]	HCC with complete remission after standard treatment modalities (n = 156)	DC-based adjuvant immunotherapy (n = 77) Control (n = 79)	RFS: No significant difference between the two groups; DC immunotherapy: in non -RFA group (n = 83) reduces recurrence risk, in RFA group (n = 61) increases recurrence risk	Serious AEs in 14.2 % of the immunotherapy group and 17.1 % in the control group
NCT02423928 [28]	Metastatic castration-resistant prostate cancer confirmed metastases and intact prostate gland (n = 18)	Cryoablation + iDC dose; Cryoablation + iDC dose + ipilimumab/pembrolizumab	mPFS: 10.5 months; mOS: 40.7 months	No grade >3 AEs
NCT04118166 [42]	Unresectable or metastatic soft tissue sarcomas (n = 30)	Ipilimumab and nivolumab for four doses, followed by nivolumab alone with cryoablation	ORR: 4 % (RECIST) and 11 % (irRECIST); mPFS: 2.7 months; mOS: 12.0 months	Grade 4 AEs in 2 patients
NCT03237572 [43]	Advanced cancer with liver metastases (n = 14)	HIFU + anti-PD-1 antibody (sintilimab/toripalimab/camrelizumab/tislelizumab)	ORR: 21.4 %; DCR: 78.6 %	No grade >3 AEs
NCT00568763 [44]	Unresectable, metastatic melanoma (n = 9)	Heat-shock therapy (42 °C, 30 min), then one of three treatments: intralesional GM-CSF, RFA + GM-CSF, or cryoablation + GM-CSF	mOS: 8.2 months	No dose-limiting toxicity
NCT03183219 [45]	HCC (n = 30) and ICC (n = 29)	Groups A (n = 15, HCC) and C (n = 15, ICC): locoregional ablation (cryoablation or IRE); Groups B (n = 15, HCC) and D (n = 14, ICC): locoregional ablation + allogeneic γδT cell transfer	Combination treatment vs. locoregional treatment groups: median distant PFS: 8 vs. 4 months (HCC), 8 vs. 4 months (ICC); OS: 13 vs. 8 months (HCC), 9.5 vs. 8 months (ICC)	No severe AEs
NCT03008343 [29]	Unresectable primary liver cancer (n = 40)	IRE group (n = 22) IRE-NK group (n = 18)	IRE-NK group vs. IRE group: mPFS: 15.1 vs. 10.6 months; mOS: 17.9 vs. 23.2 months	No severe AEs
NCT03180437 [31]	Locally advanced pancreatic cancer (n = 62)	Group A: IRE + γδT-cell infusion (n = 30) Group B: IRE (n = 32)	Group A vs. B: mPFS: 11 vs. 8.5 months; mOS: 14.5 vs. 11 months; In group A, mOS of multi-course γδT-cell infusion vs. single-course: 17 vs. 13.5 months	Grade 3–4 AEs in 14 patients
NCT03575806 [46]	Primary HCC with microvascular invasion received hepatectomy (n = 48)	T_cm_ group: central memory T-cell self-transfusion + TACE (n = 23) Control group: TACE alone (n = 25)	T_cm_ group vs. the control group: mRFS: not reached vs. 9.5 months; 1- and 2-year RFS rates: 72.0 % vs. 46.4 % and 58.18 % vs. 39.14 %	No serious AEs
NCT04174781 [47]	HCC in BCLC stage A exceeding the Milan criteria, or BCLC stage B (n = 61)	At least one treatment cycle with DEB-TACE + sintilimab	ORR: 62 % (37/60); 51 patients had undergone surgery; mPFS: 30.5 months; 12-month PFS rate among patients undergoing surgery: 76 %; pathologic CR: 14 % (7/51)	Grade ≥3 AEs in 28 % of patients
NCT04011033 [30]	Unresectable HCC after TACE failure (n = 60)	TAE (n = 30) TAE-iNKT (n = 30)	TAE-iNKT vs. TAE: mPFS: 5.7 vs. 2.7 months; ORR: 52 % vs. 11 %; DCR: 85 % vs. 33 %	Grade 3 AEs of 4 % in TAE-iNKT and 19 % in TAE
NCT03817736 [26]	Locally advanced, unresectable HCC (n = 33)	Sequential TACE and stereotactic body radiotherapy followed by avelumab	Amenable to curative treatment: 18 patients	Grade ≥3 AEs in 33 % of patients
NCT03397654 [48]	Liver-confined HCC (n = 15)	Up to two rounds of TACE followed by pembrolizumab	ORR: 53 %; mPFS: 8.95 months; mOS: 33.5 months	Grade ≥3 AEs in 46.7 % of patients
ChiCTR2100050410 [49]	Unresectable HCC (n = 55)	TACE + lenvatinib + camrelizumab	ORR: 76.4 %; DCR:85.5 %; Surgical conversion rate: 52.7 %; Radical resection rate: 96.6 %	Grade 3–4 AEs in 43.6 % of patients
NCT03380130 [50]	Unresectable HCC and liver-only disease received chemoembolization (n = 42)	SIRT followed 3 weeks later by nivolumab	ORR: 41.5 %; mTTP: 8.8 months; mOS: 20.9 months	Grade 3–4 AEs in 26 % of patients
NCT02416466 [32]	CEA^+^ liver metastases (n = 6)	Anti-CEA CAR-T hepatic artery infusions and SIRT	mOS: 8 months	No serious AEs

AEs, Adverse events; DCR, Disease control rate; HCC, Hepatocellular carcinoma; ICC, Intrahepatic cholangiocarcinoma; IRE, Irreversible electroporation; MWA, Microwave ablation; mTTP, Median time to progression; mOS, Median overall survival; mPFS, Median progression-free survival; ORR, Overall response rate; RFA, Radiofrequency Ablation; SIRT, Selective internal radiation therapy; TACE, Transarterial chemoembolization.

For instance, 668 HCC patients with high recurrence risk following surgery or local ablation were administrated with atezolizumab plus bevacizumab every 3 weeks or active surveillance (IMbrave050) [25]. Atezolizumab plus bevacizumab treatment significantly increased the RFS compared with active surveillance group, with median follow-up time of 17.4 months. Grade 3–4 AEs occurrence in atezolizumab-bevacizumab group and active surveillance group were 41 % and 13 %, respectively. To investigate the LIT-ICI combination as conversion therapy, 33 patients with locally advanced, unresectable HCC received Sequential TACE and SIRT followed by avelumab [26]. The conversion rate from unresectable to acceptable for curative treatment is 55 % (18/33). Two of the 33 patients had resection, two had RFA, and 14 had a complete radiological response and opted for close surveillance. 33 % (11/33) of the patients experienced treatment-related grade 3–4 AEs. Besides, DCs [27,28], NK cells [29], NKT cells [30], γδT cells [31], or CAR-T cell [32] infusion alone or combined with TACE or ablative therapies hold promises in amplifying cytotoxicity mediated antitumor immunity. In trial of intraarterial CAR-T cell infusion and SIRT for carcinoembryonic antigen (CEA)^+^ liver metastases, the median OS was 8 months. After hepatic artery infusions of CAR-T treatment, the levels of GM-CSF, IDO, and PD-L1 in liver metastases decreased, and the serum CEA levels of all subjects remained stable or declined. No serious treatment-related AEs occurred.

Although promising results were provided in certain types of cancer, there are substantial challenges to be addressed, including the tumor recurrence, heterogenous immune response between individuals, and the predictive biomarker of patient selection during decision-making process. The use of patient-derived organoid or ex vivo immune cell co-culture models can better evaluate efficacy and predict outcomes [33,34]. There remains key issues in the microenvironment post immune-LIT combined therapies, such as the insufficient adoptive cell infiltration into core region of tumor, local hypoxia and metabolism competition between tumor cells and lymphocytes, unexpected immunosuppressive environment (CAFs, TAM or MDSCs), and so on. Further studies should involve more specific efforts to characterize and reshape the TME post-interventional therapies.

## 3 LIT-induced immunomodulatory effects

Local ablation aims to directly destroy the target tumor lesion without damage to surrounding tissue by extremely high temperature (>60 °C), which causes unrehearsed cell injury and triggers cancer cell apoptosis or coagulative necrosis. Of note, these ablative therapies serve as antigen sources and immunostimulants for activating immune system, since tumor debris is permanently left in situ. By integrating spatial transcriptomics with histological techniques in a preclinical pancreatic cancer model, the results indicated that how the RFA and immunotherapy combination therapy reshaped TME towards an anti-tumor milieu [51]. However, tumor ablation itself is insufficient for evoking potent and robust immune responses and sometimes may cause immunosuppressive effects. Embolization therapies triggers large amounts of TAAs following ischemia-induced cell death. Soluble molecules (cytokine, chemokine, and growth factor) and immune cells (T cell, neutrophil, DC, and macrophage) are vital players in shaping TME post embolization [[52], [53], [54], [55], [56], [57]]. For example, transarterial embolization leads to the increase of CD3^+^, CD4^+^, CD8^+^, and FOXP3^+^ lymphocytes, as well as PD-L1 expression, and such influences are dependent on disease subtype, embolic agent type, and vessel distribution [58]. Currently, there is an emerging role of partial embolization for boosting immune response to larger extent instead of conventional total/complete embolization [59]. In context of high tumor burden or conversion therapy, the partial embolization facilitates anti-VEGF/TKI and/or ICI-based systemic therapy and also preserve liver function for HCC patients [60,61]. Besides, SIRT was considered as a better option to combined with immunotherapy, since its superior activation and recruitment of effector cells [62]. Therefore, understanding the immune effects of LITs on TME can empower the design of optimal and tailored strategies for precise medicine.

### 3.1 Antigen process and presentation

Compared with conventional surgical resection, interventional therapies preserve the post-treatment lesion in situ, forming an antigen-enriched hub due to the immunological cell death (ICD) effect, including necroptosis [63], pyroptosis [64], and ferroptosis [65], which plays crucial roles in altering the TME. Subsequently, danger-associated molecular patterns (DAMPs), high mobility group box 1 and ATP, are released into the extracellular tissue via the disrupted cell membrane and serve as a "find me' signal for recruiting APCs. Meanwhile, calreticulin translocates to the outer membrane of tumor cells, and functions as an "eat me' signal and facilitates APC phagocytosis of tumor associated antigens (TAAs) [66,67]. Heat shock protein 70 (HSP70) efflux under heating stress is regarded as a costimulatory signal that provokes local inflammation and triggers antitumor response. Dromi et al. found that intratumoral dendritic cells (DCs) were still detectable in the treatment group at 11 days post subtotal RFA [68]. Both RFA and MWA can enhance evident and durable TAA-specific T cell responses in HCC patients, lasting for 24 weeks [69,70]. Further clinical studies show that GPC3-expressing HCC can powerfully produce GPC3-specific CTLs after RFA/TACE but not in surgical groups [71]. Notably, by leveraging electrical pulses to disrupt cell membranes, IRE is a more efficient way to preserve the antigenicity of TAAs without heat-induced protein denaturation [72]. Kuang et al. observed that IRE induces cell necrosis and significant release of cellular molecules, including ATP, HMGB1 and calreticulin, that are vital to activate CD8^+^ T cell immunity [73]. Shimizu et al. demonstrated that IRE boosted the proliferation of tumor antigen-specific memory CD8^+^ T cells, which exerted enhanced antitumor immunity synergized with anti-CTLA-4 treatment in a prostate cancer model [74]. Furthermore, IRE is able to reverse ICI resistance by promoting the infiltration and memory establishment of CD8^+^ T cell [75]. Recently, histotripsy appears as a novel FUS tumor ablation therapy with potent immunostimulatory effects indicated in diverse preclinical models, including melanoma [76,77], HCC [78], breast cancer [79], and osteosarcoma [80]. Inoculation with cell-free tumor debris generated by histotripsy has been demonstrated potent antitumor immunity against tumor challenge [78].

Except for TAA release, thermal ablation also enforces the secretion of proinflammatory cytokines [81,82]. VEGF often overexpressed in HCC patients postRFA [83]. Interestingly, it has been demonstrated that targeting VEGF is a promising way to enhance the antitumor immune response and make ICIs more effective. Indeed, VEGF can upregulate tumor PD-1 and PD-L1 expression, and VEGF-targeted therapy can lead to transient vascular normalization that allows more T lymphocytes to infiltrate into the tumor region. The outcomes from the IMbrave 150 RCT, which enrolled 501 HCC patients, definitely demonstrated that patients in the atezolizumab plus bevacizumab group had outstanding outcomes in OS (67.2 % VS 54.6 %) and mPFS (6.8 VS 4.3 months) than the control group [84].

### 3.2 Local perfusion and metabolism

Promoting perfusion in TME is still debatable, as enhanced perfusion means that tumor cells can obtain more nutrients and energy for proliferation and have more opportunity to access blood vessels and cause hematogenous metastasis. On the one hand, hypoxia is one of the main contributors for tumor angiogenesis, growth, metastasis and failure of treatment [[85], [86], [87], [88], [89], [90]]. Embolization or ablative therapy both disrupt the intratumor vessel distribution, then leading to even more hypoxia environment which is characterized with upregulation of hypoxia-inducible factor-1α and VEGF [[91], [92], [93], [94]]. The subsequent tumor neovascularization is recognized as a critical contributor to cancer recurrence [93,95]. RFA induces the hypoxia-inducible factor-1α upregulation in preablation rim with a temperature-dependent manner [91]. The serum VEGF level is demonstrated as an independent predictor for the risk of tumor progression postTACE [96]. On the other hand, thermal therapy could improve perfusion and reduce intratumor interstitial pressure [97], which improves the accumulation of therapeutic agents or lymphocytes, and the secretion of cytokines and chemokines [[98], [99], [100]]. Albumin-bound paclitaxel (Abraxane) plus carboplatin administration after MWA treatment were investigated in lung cancer patients, which provided acceptable efficacy and safety [101]. Besides, metabolic stress in TME causes T cell exhaustion and dysfunction, especially owing to the competition for oxygen and nutrients [102,103]. Since LIT leaves a complex posttreatment TME, the dynamic metabolism should be further explored in the light of immune-metabolism crosstalk. FUS is often used to open physical barrier [[104], [105], [106]], especially blood-brain barrier, for enhancing delivery of micromolecular drug or contrast agent microbubbles into tumor.

Besides, regarding the dynamic immunometabolism in TME, metabolic reprogramming significantly influences therapeutic outcomes [107,108]. Tumor cells and immune cells both undergo metabolic changes, such as altered glycolysis and oxidative phosphorylation, affecting cell function and therapeutic response. For instance, tumor cells often exhibit increased glycolysis, known as the Warburg effect, supporting rapid proliferation. This shift can lead to the accumulation of lactate, creating an acidic environment that suppresses immune cell activity. Conversely, immune cells in the TME may experience metabolic constraints, limiting their effector functions. As we all known, LITs induce profound alterations in the tumor metabolic microenvironment, which disrupt local blood flow and oxygen supply, leading to acute hypoxia and ischemia. Consequently, there is a metabolic shift from oxidative phosphorylation to anaerobic glycolysis, accompanied by lactate accumulation and acidosis. Furthermore, the deprivation of nutrients and oxygen alters lipid and amino acid metabolism, promoting a stress-adaptive metabolic phenotype. Targeting these metabolic pathways post LITs offers potential therapeutic strategies to enhance treatment efficacy.

### 3.3 Physical barrier and cell infiltration

There is an unexpected firewall established between tumor core area and precancerous immune niche, which hinders the cytotoxic T lymphocytes infiltration. In HCC context, cross-presentation of antigens appears between pericancerous macrophages and CD103^+^ CTLs, causing the CD103^+^ CTLs accumulation in the pericancerous area, then promoting tumor progression as well as immunotherapy resistance [109]. This barrier phenomenon could be even more common post-thermal ablation, since the heat-induced cell damage is not restricted to cancer cells; other cell types, such as stromal cells and fibroblasts, are also involved. Cancer-associated fibroblasts (CAFs) can develop a "physical barrier', produce protumoral extracellular matrix proteins and cytokines and prevent CTLs from migrating into the tumor region, which results in a desmoplastic and immunosuppressive TME and impedes the efficacy of ICIs and chemotherapy [110]. It has been reported that targeting Pin1 on CAFs can reduce collagen deposition, tumor growth, and CAF activation and proliferation by inhibiting lysosomal degradation of PD-L1 and ENT1 in tumor cells and activating other cancer-associated pathways [111]. In contrast, certain benefits are from direct thermal denaturation, wherein the peripheral thermal region of the lesion experience a rim of acute inflammatory response leading to immune cell recruitment. Additionally, increased blood flow in the periphery of the thermal region not only alters the hypoxic state and sensitizes the tumor tissue to antitumor therapies, but also makes the nanomaterials accessible to tumors.

### 3.4 Suppressive effect

In some cases, subtotal ablative therapy accelerates residual cancer progression and confront with poor prognosis [[112], [113], [114], [115]]. To reveal the underlying mechanism, Kuang's group investigated and reported a series work [34,[116], [117], [118]]. Thermal stimulation obviously activates the transcription factor SP1 via mechanical stress, which increases IL4-Induced-1expression, and catalyzes tryptophan metabolites to activate the aryl hydrocarbon receptor [116]. This could be a significant mechanism for cancer invasiveness postRFA. Epigenetic modifications on various RNA species also participate in cellular dynamic response and final fate to heat stress [119,120]. Sublethal thermal stress increases the m^7^G tRNA [34] modification and m^6^A mRNA modification [117], and targeting the corresponding axis may prevent tumor progression. Preclinical evidence also indicated a more suppressive environment featured excessive myeloid suppressor cells, tumor-associated macrophages (TAMs), and tumor CCL2 secretion after incomplete RFA, which in turn hindered the anti-PD-1 immunotherapy [121]. Targeting the METTL1-TGF-β2-PMN-MDSC axis was to remodel TME and reduce HCC relapse postRFA [118]. Besides, Li's group characterized the immune landscape following TACE, and TREM2^+^ TAMs were observed to apparently suppress CD8^+^ T cell proliferation, which demonstrated the significance of TREM2 deficiency for the therapeutic improvement of anti-PD-L1 immunotherapy [122]. Ueshima et al. revealed that TAE increases the intratumor immunosuppressive TGF-β1 expression in the rat hepatoma model, and targeted inhibition of TGF-β1 could extend animal survival [123].

### 3.5 Abscopal effect

Immunomodulation effect in systemic landscape triggered by interventional therapy was also confirmed in distant, untreated tumors. Increased serum levels of proinflammatory factors HSP70 [124,125], Interferon (IFN)-γ [69], TNF, IL-6, and reduced anti-inflammatory cytokines (TGF-β, IL-10) were detected in RFA-treated malignant tumor [81]. In a spontaneous breast cancer lung metastasis model, primary tumor ablation inhibited lung metastasis via activation of the macrophage-IL15-NK cell axis [126]. Similarly, patients who underwent a better NK cell response after RFA were correlated with better disease-free survival [127]. Wang's group unveiled the MWA-induced systemic immune response in 6 breast cancer patients [128]. Single-cell RNA sequencing indicated the peripheral activated NK and CD8^+^ T cells, enhanced co-stimulatory signature of CD4^+^ T cells, as well as increased ICOS^+^CD4^+^ T cells were observed after MWA treatment. Recently, abscopal effect is often observed after cryoablation-treated patients [[129], [130], [131]]. In lung cancer patients, cryoablation significantly increases peripheral CD8^+^ T cell subpopulations and IFN-γ expression [132]. In mouse cancer models, the STING-dependent type I IFN signaling pathway was mechanistically responsible for enhanced systemic immune response [132,133]. Combined therapy with cryoablation and anti-PD-1 blockade could augment the infiltration of CD8^+^ T cells, CD4^+^ T cells, DCs and M1-like TAMs, and reduce immunosuppressive M2-like TAMs and MDSCs in distant tumors [134]. The U.S. FDA newly-approved non-thermal histotripsy treatment also induced potent abscopal effect on preclinical melanoma or HCC [76,78]. Notably, a safety and efficacy clinical trial of histotripsy for liver tumor reported an abscopal effect, which involves a reduction in the volume of nontreated tumor lesions in liver, and a sustained reduction of tumor biomarker (CEA) [135]. This compelling results promotes future clinical validation in large-scale population.

## 4 Rationally designed immuno-nanostrategies synergize with LIT

### 4.1 Biomaterials promote in situ vaccine

Tumor thermal ablation is one of the most common LITs for solid tumor in clinical settings. Hyperthermia therapy based on photothermal agents or nanoparticles is often performed on superficial tumors of patients or pre-clinical animal models. Embolization could induce similar ICD via chemotherapeutic agents or starvation strategy [136]. These heating or embolization treatments not only initiate in situ vaccination effect, but also triggers the release of tremendous proinflammatory cytokines and chemokines. Compared to the exogenously delivered tumor vaccine, such as live-attenuated vaccines [137,138], mRNA vaccines [[139], [140], [141]], or recombinant vector vaccines [142,143], the in situ tumor vaccine possesses integrated antigen resource including neoantigen, which holds promises in triggering potent and prompt antitumor response. However, this immunoactivation is insufficient and not durable enough to fight against residual tumor cells or even tumor recurrence. Bearing this in mind, current nanostrategies are developed and administered post LIT, aiming to inhibit tumor recurrence via amplifying in situ vaccination effect.

As a typical example, Li et al. constructed the bisphosphonates with dual electric potentials (BNV(+&−)) [144]. The proposed nanovaccine enabled effective capture of various released tumor pan-antigens postRFA. Bisphosphonate also acted as an immunoadjuvant by blocking mevalonate metabolism in TME. Furthermore, BNV(+&−) loaded with neoantigens was injected subcutaneously to activate systemic immunity. This novel strategy triggered spatiotemporal immune effect and provided precise delivery of tumor vaccines. To reduce the postRFA metastases and recurrence by adding immunoadjuvants, Tian et al. proposed an in situ nanovaccine formed by layered double hydroxides encapsulating a STING agonist, cGAMP, denoted as LDHs-cGAMP, which adsorbed tumor antigens to potentiate antitumor immune response [145]. Additionally, virus-derived nanoparticles could be alternative adjuvant for immunomodulation. Ghani et al. combined cryoablation and cowpea mosaic virus-derived nanoparticles as an immunoadjuvant, which was extracted from plant virus containing a single-stranded RNA genome [146]. In subcutaneous RIL175 cell-derived HCC models, cryoablation plus CPMV treatment exerted excellent tumor inhibition and abscopal effect.

**Fig. 2:**
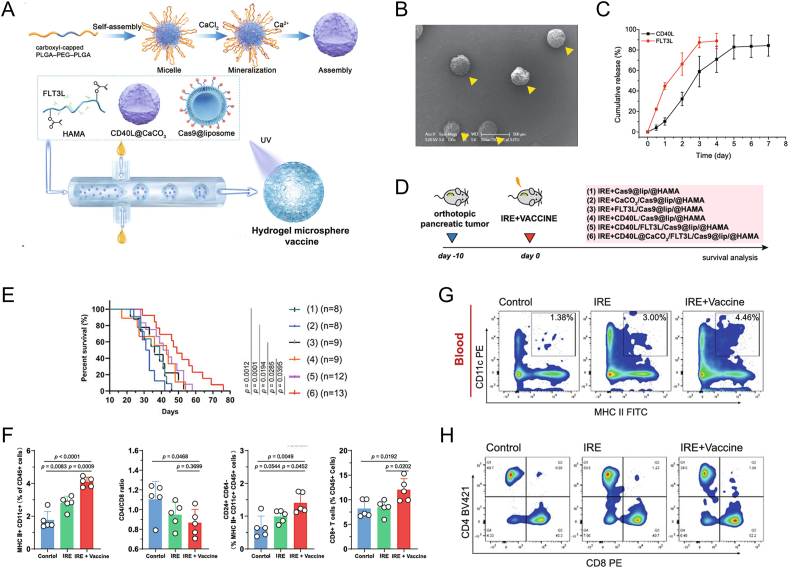
**Interventional hydrogel microsphere vaccine as an immune amplifier for activated antitumor immunity after IRE.** The hydrogel microsphere vaccine improved the recruitment and amplification of tumor-resident cDC1s by rapidly releasing FLT3L, followed by the controlled release of CD40L in the acidic TME, which further amplified cDC1 maturation and migration into tumor-drained lymph nodes. A) Schematic of the hydrogel microsphere vaccine preparation. B) Scanning electron microscope image of the hydrogel microspheres. C) The *in vitro* release profile of FLT3L and CD40L from the hydrogel microspheres. D) Schematic of combined therapies in pancreatic cancer model. E) Animal survival curve of indicated treatment. F) The hydrogel microsphere vaccine increased the circulating CD11c^+^MHC II^+^ cells, CD8^+^ T-cell-to-CD4^+^ T-cell ratio, CD24^+^CD64^−^ cells, and CD8^+^ T cells. G,H) Flow cytometry analysis showing the proportion of circulating CD11c^+^MHCII^+^ cells (G) and CD8^+^ T cells (H). Data are presented as mean ± SEM. The log-rank (Mantel–Cox) test for survival curves was used in E, and two-tailed unpaired *t*-test was used in F. Reproduced with permission [151]. Copyright 2022, Springer Nature.

As APCs play vital roles in antigen process and presentation, emerging studies have made efforts to strengthen the antigen recognition, phagocytosis behavior of DCs and the following presentation to cytotoxic T cells. To capture the cryoablation-generated tumor fragments and achieve lymph node targeting, Hu et al. synthesized maleimide-modified nanoparticles encapsulating Astragalus polysaccharide, which both suppressed primary tumor and secondary recurrence as an in situ nanovaccine [147]. Integrated with toll-like receptor 9 agonists (CpG oligodeoxynucleotide, CpG-ODN), the nanoadjuvant could be effectively internalized by APCs and synergized with IRE in MR and CT imaged theranostics [148]. HSPs are released from tumor cells following thermal ablation-induced stress or necrosis. These molecular chaperones can bind to TAAs and serve as DAMPs, which are recognized by pattern recognition receptors on DCs. The interaction promotes DC maturation, enhances antigen processing and presentation, and facilitates the activation of tumor-specific T-cell responses. Accordingly, Zhou et al. developed a mannose-modified carbon dots (Man-CDs), which absorbed postMWA "danger signals' (such as HSPs or other TAAs) in situ and then targeted deliver to DCs [149]. The *in vivo* animal experiment showed that postMWA injection of Man-CDs triggered a potent tumor-specific immune response and effectively hindered the hepa 1-6 tumor growth. In addition, Chen's group reported a Rho-associated kinase (ROCK) blockade-hydrogel, which aimed to promote DC phagocytosis postRFA. ROCK inhibitor, Y27632, is first dispersed in PLGA-PEG-PLGA solution in vitro, which forms hydrogel quickly in vivo, obviously prolonging the retention of Y27632 [150]. With improved DC phagocytosis of antigens, cytotoxic T cells are further activated for potent antitumor immune responses. Wang and co-workers reported a hydrogel microsphere loaded with FLT3L and CD40L as a tumor vaccine combined with cryoablation, which facilitates cDC1 migration to the lymph nodes, leading to enhanced CD8^+^ T cell cytotoxicity in pancreatic cancer (Fig. 2) [151]. The altered expression of HMGB1, TNF-α, IFN-γ, and PD-L1 were attributed to the immunostimulation via injectable hydrogel vaccine, which holds satisfied biocompatibility and safety *in vivo*. Overall, advanced strategies of in situ nanovaccine showed potent efficacy in preclinical models, further clinical translation is still urgently in need.

### 4.2 Biomaterials enhance ICIs

Although immune checkpoint blockade has achieved great progress in a series of clinical trials, only a few patients could achieve durable survival benefits. Emerging clinical evidence suggested that the efficacy of PD-1, PD-L1, or CTLA-4 blockade are different among patient classification (tumor subtype, disease stage, receptor expression, administration combinations, etc.). LIT thereby offers a synergistic contribution to the local and systemic immunostimulation, in terms of reducing tumor burden and triggering abscopal effect. With enhanced and precise targeting behavior, nanomaterials exactly provide significant bridge between LIT and ICI to maximize the treatment efficacy. Besides, considering the enhanced tumor accumulation of ICIs, nanomaterials could also reduce the off-target effects and possible AEs in clinic, lead to prolonged treatment window for patients. The summarized related studies are listed in Table 2. The nanomaterial-based LIT and immune checkpoint blockade therapy mainly categorized into three aspects: 1). Nanoparticles to targeted delivering ICIs [[152], [153], [154]]; 2). Nanoparticles to promote LIT efficacy and then combined with ICI administration [[155], [156], [157], [158], [159], [160]]; 3). Nanoparticles to targeted inhibition of immune checkpoint expression [161,162].

**Table 2:** A brief summary of targeting immune checkpoints nanomaterials for synergized LITs.

Targeting immune checkpoint	Nanomaterial	LIT	Tumor model	Outcomes	Ref
Delivery of anti-PD-L1 antibody	Platelets@anti-PD-L1	HIFU, PDT, radiotherapy	4T1 breast cancer mouse model	Increased the delivery of anti-PD-L1 to tumor residues	[152]
Delivery of anti-PD-L1 antibody	Hollow mesoporous manganese dioxide nanoparticles encapsulating FIDAS-5 with macrophage membrane coating and surface modification of anti-PD-L1	RFA	H22 liver cancer mouse model	Augmented HCC antigenicity to stimulate cytotoxic T cell recognition and cytotoxic killing, mediated immunogenic cell death	[153]
Delivery of anti-PD-1 antibody	PLGA nanoparticle encapsulating imiquimod and coated with PD-1 expressed cell membrane	IRE	CT26 colon cancer model	Generated potent and systemic anti-tumor immune response, remarkably suppressed distant tumors	[154]
Delivery of anti-PD-1 antibody	Nivolumab loaded silk embolic hydrogel	TAE	Porcine arterial embolization model	Achieved embolization of porcine arteries without recanalization and delivered both albumin and Nivolumab	[165]
Combined with PD-1 inhibition	Silica microshell loaded with perfluorocarbon liquid + anti-PD-1 i.p. injection	HIFU	GL261 glioblastoma mouse model	Induced a "hot' immune-microenvironment and achieved tumor remission	[155]
Combined with PD-1 inhibition	Hemin & lipoxidase co-loaded CaCO_3_-encapsulated PLGA + anti-PD-1 i.v. injection	RFA	4T1/H22/human PDX tumor mouse model; VX_2_ rabbit tumor model	Inhibited the growth of both residual and metastatic tumors	[156]
Combined with PD-L1 inhibition	Superparamagnetic CoFe_2_O_4_@MnFe_2_O_4_ + anti-PD-L1 i.v. injection	MHT	4T1 breast cancer mouse model	Produced numerous tumor-associated antigens for effective immunotherapy of distant metastatic tumors	[157]
Combined with PD-L1 inhibition	Metallic supra-structured cryo-nanocatalyst + anti-PD-L1 i.p. injection	Cryoablation	TrampC1 prostate cancer mouse models; MCA-RH7777 HCC rat model	Promoted ice formation and necroptosis, enhanced anti-tumor immune responses	[158]
Combined with CTLA-4 inhibition	Chlorine e6 loaded mesoporous organosilica bodies with magnetic heads + anti-CTLA-4 i.v. injection	MHT, PDT	MCF-7 or 4T1 breast cancer mouse model	Eradicated primary and metastatic tumors with low systematic toxicity	[159]
Combined with CTLA-4 inhibition	R837@PLGA or MPLA@PLGA + anti-CTLA-4 i.v. injection	RFA, HIFU	CT26 colon cancer mouse model	Induced robust tumor vaccine-like responses to inhibit metastatic tumor and recurrence via anti-tumor immune responses	[160]
Silencing PD-L1 expression	Camptothecin &PD-L1 siRNA CRNPs	Cryoablation	EO771 or 4T1 breast cancer model	Induced strong immunogenic cell death, promoted maturation of DCs, and activation of CD8^+^ cytotoxic T cells and memory T cells	[161]
Amlodipine triggered the autophagic degradation of PD-L1 in exosome	Polydopamine loaded with GW4869 & amlodipine	RFA	Hepa1-6 and H22 liver tumor model; Orthotopic N1-S1 HCC Rat Models	Remodeled TME substantially,inhibited the progression and metastasis of HCC	[162]

For example, nanoparticle-mediated mild magnetic hyperthermia therapy can induce antitumor immune response involving macrophages, DCs, and T cells [163,164]. To further optimize the magnetic hyperthermia related immune performance, Pan et al. synthesized superparamagnetic CoFe_2_O_4_@MnFe_2_O_4_ nanoparticles for magnetic hyperthermia treatment under an alternating magnetic field combined with α-PD-L1 intravenously injection [157]. This combination therapy can not only entirely ablate primary tumors but also generate TAAs presented by DCs to stimulate native T cells. Under treatment with α-PD-L1, the suppressive T cells were relieved; therefore, a large amount of cytotoxic T cells infiltrated into the distant tumor. Incomplete RFA was considered as a critical contributor to tumor progression post ablation, a nanostrategy of targeting PD-L1 expression of tumor exosome, which integrated GW4869 and amlodipine into a polydopamine nanoparticle [162]. GW4869 and amlodipine enables exosome secretion and triggered PD-L1 autophagic degradation, collectively remodeling immune environment and conquering tumor metastasis post RFA. To achieve transarterial delivery of anti-PD-1 antibody into tumor, Oklu et al. developed a novel silk protein-based embolic hydrogel encapsulating nivolumab and confirmed acceptable embolization efficacy in healthy porcine model [165]. Further evaluation is expected using a cancer model which receives embolization and PD-1 blockade combined therapy.

### 4.3 Biomaterials improve cell-based immunotherapy

ACT is explored to leverage natural or genetically engineered T cells, NK cells, NKT cells and macrophages isolated from patients to specifically eliminate cancer. However, the potency of ACT against solid tumors remains insufficient due to the limited tumor-specific antigens, dense ECM, and immunosuppression environment. These factors substantially impair the intratumoral infiltration, proliferation, and capacity of adoptive cells.

**Fig. 3:**
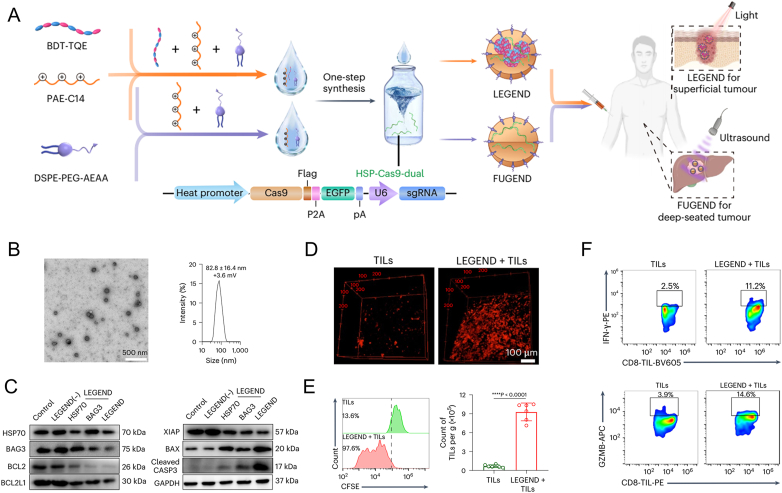
Non-invasive activation of intratumoral gene editing for improved TILs in solid tumors. A) Schematic of LEGEND or FUGEND design for non-invasive intratumoral gene editing. B) Transmission electron microscopy image and size distribution of prepared nanoparticles for LEGEND usage. C) Western blot analysis of HSP70, BAG3, anti-apoptotic proteins (BCL2, BCL2L1, XIAP) and pro-apoptotic proteins (BAX, cleaved CASP3) in LEGEND-treated B16F10 cells. D) increased TILs after LEGEND treatment. E) Enhanced proliferation of TILs after LEGEND treatment. F) Flow cytometry displaying the percentages of IFN-γ^+^ and GZMB^+^ in activated CD8^+^ TILs. Data are presented as mean ± SD. Two-tailed unpaired *t*-test was used in E. Reproduced with permission [166]. Copyright 2023, Springer Nature.

Instead of directly eliminating tumor, mild heating sometimes could act as an external trigger for modulating TME. Using non-invasive near-infrared light or FUS as a heat generator, Ping et al. reported a temperature-responsive nanodevice for Cas9 gene editing in tumor cells to reduce the apoptosis resistance, denoted as LEGEND or FUGEND (Fig. 3A) [166]. Specially, the LEGEND was prepared by coassembly with poly(β-amino ester) (PAE-C14), semiconducting polymer (BDT-TQE), tumor-targeting PEGylated lipids (DSPE-PEG-AEAA) and a heat-inducible Cas9 plasmid encoding sgRNA of HSP70 and BAG3 (HSP-Cas9-dual). These round-shaped nanoparticles could induced apoptotic gene up-expressed in tumor cells (Fig. 3B and C). In tumor-bearing model, the infiltration, proliferation and cytotoxicity of TILs were also enhanced after LEGEND treatment, indicating the immunomodulation of LEGEND strategy (Fig. 3D–F). By disrupting the physical barriers, this can modulate extracellular TME and facilitate adoptive T cell infiltration. Similarly, under a HSP promote control r, FUS-generated heat was able to improve the genetics and cellular functions of CAR-T cells within tumors [167]. This acoustogenetic manipulation on engineered T cells offers a novel prospective on cell-based therapies. The therapeutic efficacy of adoptively transferred natural killer T (NKT) cells is hindered by insufficient tumor infiltration and inadequate activation. To overcome these obstacles, Wang et al. developed a PBIBDF-BT encapsulated with PLGA nanoparticle (NPs@PBT), to achieve photothermal therapy (PTT) [168]. Owing to PTT-induced inflammation, especially chemotaxis recruitment and complement triggered vasodilation, the infiltration of NKT cells in tumor were remarkably elevated and the NKT cell-based immune cascade were initiated. The combined therapy of PTT plus NKT cell transfer demonstrated obvious growth inhibition of both local and distant tumors, as the result of long-term immunological memory. The conventional ACT therapy targeting solid tumors, mainly based on intravenous injection of *in vitro* expanded cells. Considering the difficulty of in homing capability to tumor, using biomaterials to deliver adoptive cells via intertumoral injection may serve as an alternative. Park et al. encapsulated NK cells into 3D bioprinting hydrogels via thermally sensitive gelatin to form cell containing micro/macropore [169]. After implanted hydrogel into the tumor site, cell viability, cytotoxic effect, and cytokine release of NK cells were enhanced. In addition to enhance blood vasodilation and permeability, thermal effect on the physical barrier such as dense exocellular matrix and stroma, also contribute to TILs penetration. Tao et al. proposed the tin monosulfide nanoparticles (SnSNPs) that can overcome the stromal barrier [170]. Upon near-infrared irradiation, mild photothermal effect of SnSNPs allowed in situ tumor collagen denaturation and deep penetration of cytotoxic T lymphocytes into the tumor.

Tough some cases have tried transarterial infusion of NK cells [171] or GPC3^+^ CAR T cells [172] in HCC patients with satisfying outcomes, the EPR effect of nanomaterials may provide additional value for targeted cell delivery while overcoming physical barrier; such further studies on are urgently in need.

### 4.4 Biomaterials modulate TME

The LIT itself and LIT induced immune alternation in TME are both dynamic and complex process, companied by various immune cells and metabolic pathways. The unexpected immunosuppression is markedly associated with limited efficacy and poor prognosis. For instance, uncontrollable heat diffusion in TME has been a long-unaddressed problem which contributes to the immunosuppression and tumor recurrence post thermal ablation. To this end, Zhang et al. established a in situ Au bioreactor in thermally sensitive hydrogel, which was intratumorally injected in advance for better heat dispersion during MWA treatment [173]. Subsequently, the valid ablation zone was enlarged and extracellular matrix was also reprogrammed at the same time. Various immune cells could be easily activated or infiltrated into tumor, such as DCs, NK cells, M1 macrophages, and CD8^+^ T or CAR T cells.

MDSCs remain an important player in the formation of immunosuppressive TME, and unfortunately local ablative therapies often induce increased MDSCs. In the nonlethal transition zone of incomplete RFA or MWA, the upregulated MDSCs feature high PD-L1 expression leading to immune evasion. Thus, a novel size-tunable nano-microliposome was developed to co-deliver anti-PD-L1 antibodies and MDSC inhibitors to activate antitumor immune responses [174]. Similarly, the MDSC inhibitors was encapsulated into hydrogel along with oxaliplatin to target MDSCs post MWA [175]. Meng et al. proposed another strategy which leverage H_2_S release in situ to inhibit MDSC accumulation in TME, by constructing a Bi-MOF nanostructure, which release L-cysteine to react with the highly expressed cystathionine β-synthase in tumor to generate H_2_S [176]. This Bi-MOF can also scavenge ROS, further reversing the MDSCs-mediated immunosuppression post MWA.

TAMs consist of pro-inflammatory M1 and anti-inflammatory M2 subtype, and the altered M1/M2 ratio directly relates to tumorigenesis and progression. Park and co-workers synthesized a CpG-ODN-coated Mn-phenolic nanoparticle in combination with IRE for improved cancer immunotherapy. The proposed nanoparticles are effectively internalized into TAMs, and successfully trigger M1 polarization via TLR9 and cGAS/STING pathway activation, ultimately promoting release of proinflammatory DAMPs, cytokines (IL15, IL18, TNF) and chemokines (CXCL13, CXC15, CCL5, CCL9) [177].

The abnormal indolamine 2,3-dioxygenase 1 (IDO1) metabolic pathway represents a vital contributor to generate immunosuppressive kynurenine from tryptophan, which further result in Tregs accumulation and CD8^+^ T cell exhaustion. The proposed IDO1 inhibitors loaded iron-oxide-nanocube clusters enabled effective IRE treatment and TME modulation for tailored therapeutic strategy [178]. Another IDO-1 signaling pathway inhibitor NLG919 was also delivered via a MOF nanostructure to suppresses IDO-1 activity and reverse immunosuppression for improved HIFU treatment [179].

**Fig. 4:**
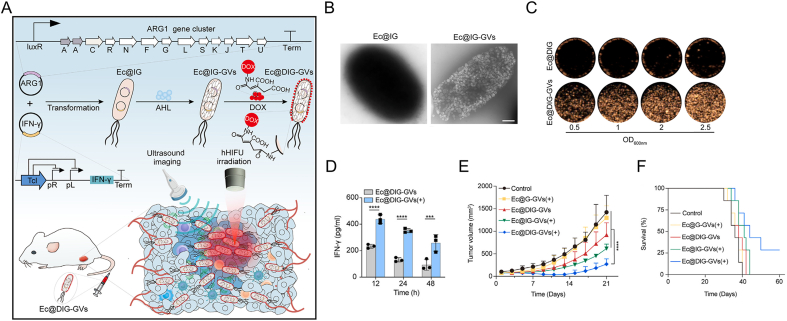
**Ultrasound-visible engineered bacteria for tumor chemo-immunotherapy.** A) Schematic of genetically engineer Ecoli, with an acoustic reporter gene and the hyperthermia-responsive IFN-γ gene, and loaded with DOX. B) TEM images of GVs in Ec@IG-GVs. C) CEUS images of Ec@DIG and Ec@DIG-GVs *in vitro*. D) The concentrations of tumor IFN-γ level at indicated timepoints with or without hHIFU treatment. Tumor growth E) and animal survival F) of 4T1 tumor models received indicated treatments. Two-way analysis of variance with Tukey's test in H. Data are presented as mean ± SD. Reproduced with permission [181]. Copyright 2024, Cell Press.

IFN-γ holds potent immunoactivation effect on various antitumor response [180]. Yan and co-workers reported an ultrasound-visible engineered *Ecoli* with the HIFU radioation triggered acoustic response in CEUS, and also modified DOX outside, denoted as Ec@DIG-GVs (Fig. 4) [181]. The Ec@DIG-GVs can produce gas vesicles (GVs) with a real-time imaging guidance for remote hyperthermia HIFU to upregulate IFN-γ gene expression. IFN-γ improved M1 macrophage polarization and DC maturation in several tumor models.

To reverse the tumoral immunosuppressive phenotype, Ranjan et al. combined histotripsy and a ICD-enhancing Calreticulin-Nanoparticles (CRT-NPs) in the local treatment of poorly immunogenic mouse oral squamous carcinoma, and also observed gut microbiome change as the predictive biomarkers of immunomodulatory effect [182]. Besides, anti-angiogenic drugs primarily inhibit the formation of tumor neoangiogenesis by the blockade of VEGF/VEGFR signaling pathway. Beyond depriving tumors of oxygen and nutrients, targeting VEGF also normalize the abnormal tumor vasculature, improve perfusion and alleviate hypoxia. This normalization facilitates the infiltration of immune effector cells and also enhances the delivery of immunotherapeutic agents. Additionally, anti-angiogenic therapy can reverse VEGF-mediated immunosuppression by promoting DC maturation, reducing Tregs and MDSCs, and restoring cytotoxic T cell activity, thereby amplifying systemic antitumor immunity. Although previous studies proposed nanostrategies targeting VEGF via hydrogel, gold nanoparticles, or manganese dioxide nanoparticles-based drug delivery [[183], [184], [185]], these nanomaterials are expected to combine with LITs to further evaluate synergistic effects. Collectively, nanomaterials act as significant immunomodulators for reshaping suppressive TME into more activated status for better immune-LIT combinations.

In summary, the comparative analysis of different nanomaterials used in LITs for immunomodulation were listed in Table 3. Several examples of Inorganic, organic, carbon-based, biomimetic, and hybrid NPs were included, highlighting key aspects such as category, function, biocompatibility, and efficacy in immunomodulation.

**Table 3:** Comparative analysis of different nanomaterials used in LITs.

Category	Nanoparticles (NPs)	Function	Biocompatibility	Efficacy in immunomodulation
Inorganic NPs	Mn-based metal NPs	Mn as STING agonist	Safe with suitable concentration	Activate cGAS/STING pathway
	Layered double hydroxides NPs	Nanocarrier for STING agonist delivery & adsorb tumor antigens	Biocompatible, with minimal inflammatory or cytotoxic responses	Activate cGAS/STING pathway
	Iron-oxide-nanocube clusters	Nanocarrier for IDO inhibitor delivery	FDA-approved	Decrease Tregs & promote CD8^+^ T cell accumulation
Organic NPs	Hydrogel microsphere	Nanocarrier for FLT3L & CD40L delivery	Highly biocompatible, especially when composed of FDA-approved synthetic polymers	Promote DC maturation & antigen presentation
	PLGA NPs	Nanocarrier for PPT agent delivery	FDA-approved	Promote adoptive cell infiltration into tumor
	Polydopamine NPs	Nanocarrier for autophagy agonist delivery	Considered safe with proper surface modification & dosage control	Trigger the autophagic degradation of PD-L1
Carbon-based NPs	Carbon dots	Capture TAAs post MWA	Relatively safe with less inflammation or fibrosis	Target DC presentation
Biomimetic NPs	Plant-derived NPs	Immunoadjuvants	High compatibility	Increase in situ vaccination effect
	Platelets	Carrier for ICI delivery	High compatibility	Increase the targeted delivery to residual tumor
Hybrid NPs	Mn-phenolic NPs	Nanocarrier for CpG-ODN delivery	Favorable biocompatibility due to natural composition, biodegradability, & low immunogenicity	Trigger M1 polarization

## 5 Conclusions and future prospects

Currently, cancer-related death continues to be one of most severe public health issue worldwide. Compared with traditional surgery, chemotherapy, or radiotherapy, LITs possess its own advantages of microinvasive procedure and immunomodulatory effects. Immune-LIT combined therapies are promising clinical strategies for solid tumors, considering personalized and tailored cancer strategy. The difficulty to modulate antitumor immunity in TME is the high tumor heterogeneity and dynamic immune landscape. Notably, the diversity of nanomaterials can meet various immune features, so immunomodulating nanomaterials with excellent biocompatibility have been under wide and deep investigations, offering certain promising results.

However, formidable challenges still remain, hindering the broader application of these nanostrategies. Some considerations on the application and optimization of biomaterials synergized immune-LIT combinations are proposed as follows.

(1) First, the cytotoxicity and biocompatibility of nanoparticles is essential, especially given the emphasis on their clinical potential. Machine learning models coupled with high-throughput *in vitro* bioassays were reported to develop for predicting the toxicity of metal oxide nanoparticles in immune cells [186]. Similarly, when screening and evaluating the nanomaterials to assist LIT-immunotherapy, the integration of artificial intelligence technologies could contribute to efficient and robust selection of safe nanomaterials.
(2) In addition to the safety assessment of nanomaterials, the scalability and regulatory challenges is essential for translating preclinical success of nanostrategies into clinic. The key manufacturing challenges involves batch-to-batch consistency, scale-up of multifunctional nanomaterials, and the need for standard manufacturing guideline. Future studies should focus on novel technologies such as modular nanoparticle design, AI-assisted formulation optimization, and continuous microfluidic method.
(3) Another major challenge is the biological heterogeneity between animal models and patients. Differences in immune system composition, metabolic rates, and disease progression often lead to discrepancies in therapeutic responses. The use of patient-derived organoid or humanized murine models may be better to evaluate efficacy and predict outcomes in future studies. These are indispensable tools for enhancing the translational value of preclinical research and accelerating the development of clinically effective therapies.
(4) Last but not the least, which kind of LIT is best for combination, or when is the optimal timing for LIT or immunotherapy administration was still vague and need more reliable clinical trials to explore. For instance, in preclinical study designs, the use of time-staggered administration models and longitudinal immune profiling could help to elucidate the temporal dynamics of therapeutic synergy. In addition, potential randomized controlled trial may be performed aiming to compare several LITs-immunotherapy combinations, or different timepoints setup of administration.

In conclusion, biomaterials are rapidly developing to benefit immune-LIT combinations, and have shown a great success in various preclinical models. With continuous optimization, the promotion of clinical translation will ultimately benefit patients by directing disease diagnosis and treatment.

## CRediT authorship contribution statement

**Ting Luo:** Writing – review & editing, Writing – original draft, Conceptualization. **Kunpeng Ma:** Writing – original draft, Conceptualization. **Yi Zhang:** Writing – original draft, Data curation. **Qingwen Xue:** Visualization, Data curation. **Jie Yu:** Supervision. **Xing-Jie Liang:** Supervision. **Ping Liang:** Validation, Supervision.

## Ethics approval and consent to participate

This review article does not require any ethical approval or allied consent for publication.

## Declaration of competing interest

The authors declare no competing interests.

## References

[1] Llovet J.M., De Baere T., Kulik L., Haber P.K., Greten T.F., Meyer T., Lencioni R. (2021). Locoregional therapies in the era of molecular and immune treatments for hepatocellular carcinoma. Nat. Rev. Gastroenterol. Hepatol..

[2] Palmer D.H., Malagari K., Kulik L.M. (2020). Role of locoregional therapies in the wake of systemic therapy. J. Hepatol..

[3] Duan Y., Zhang H., Tan T., Ye W., Yin K., Yu Y., Kang M., Yang J., Liao R. (2024). The immune response of hepatocellular carcinoma after locoregional and systemic therapies: the available combination option for immunotherapy. Biosci Trends.

[4] Daver N., Alotaibi A.S., Bücklein V., Subklewe M. (2021). T-cell-based immunotherapy of acute myeloid leukemia: current concepts and future developments. Leukemia.

[5] Vago L., Gojo I. (2020). Immune escape and immunotherapy of acute myeloid leukemia. J. Clin. Investig..

[6] Rodriguez-Otero P., Usmani S., Cohen A.D., van de Donk N., Leleu X., Gállego Pérez-Larraya J., Manier S., Nooka A.K., Mateos M.V., Einsele H., Minnema M., Cavo M., Derman B.A., Puig N., Gay F., Ho P.J., Chng W.J., Kastritis E., Gahrton G., Weisel K., Nagarajan C., Schjesvold F., Mikhael J., Costa L., Raje N.S., Zamagni E., Hájek R., Weinhold N., Yong K., Ye J.C., Sidhana S., Merlini G., Martin T., Lin Y., Chari A., Popat R., Kaufman J.L. (2024). International Myeloma Working Group immunotherapy committee consensus guidelines and recommendations for optimal use of T-cell-engaging bispecific antibodies in multiple myeloma. Lancet Oncol..

[7] Sperling A.S., Anderson K.C. (2021). Facts and hopes in multiple myeloma immunotherapy. Clin. Cancer Res..

[8] Susek K.H., Schwietzer Y.A., Karvouni M., Gilljam M., Keszei M., Hussain A., Lund J., Kashif M., Lundqvist A., Ljunggren H.G., Nahi H., Wagner A.K., Alici E. (2023). Generation of NK cells with chimeric-switch receptors to overcome PD1-mediated inhibition in cancer immunotherapy. Cancer Immunol. Immunother..

[9] Ansell S.M. (2019). Immunotherapy in hodgkin lymphoma: the road ahead. Trends Immunol..

[10] Chu Y., Lamb M., Cairo M.S., Lee D.A. (2022). The future of natural killer cell immunotherapy for B cell non-hodgkin lymphoma (B cell NHL). Curr. Treat. Options Oncol..

[11] Ansell S.M., Lin Y. (2020). Immunotherapy of lymphomas. J. Clin. Investig..

[12] Hassanzadeh A., Rahman H.S., Markov A., Endjun J.J., Zekiy A.O., Chartrand M.S., Beheshtkhoo N., Kouhbanani M.A.J., Marofi F., Nikoo M., Jarahian M. (2021). Mesenchymal stem/stromal cell-derived exosomes in regenerative medicine and cancer; overview of development, challenges, and opportunities. Stem Cell Res. Ther..

[13] Galluzzi L., Aryankalayil M.J., Coleman C.N., Formenti S.C. (2023). Emerging evidence for adapting radiotherapy to immunotherapy. Nat. Rev. Clin. Oncol..

[14] Xu Z., Khokhlova T.D., Cho C.S., Khokhlova V.A. (2024). Histotripsy: a method for mechanical tissue ablation with ultrasound. Annu. Rev. Biomed. Eng..

[15] Schuster T.G., Wei J.T., Hendlin K., Jahnke R., Roberts W.W. (2018). Histotripsy treatment of benign prostatic enlargement using the vortx R(x) system: initial human safety and efficacy outcomes. Urology.

[16] Bogdanovic A., Djokic Kovac J., Zdujic P., Djindjic U., Dugalic V. (2023). Liver resection versus transarterial chemoembolisation for the treatment of intermediate hepatocellular carcinoma: a systematic review and meta-analysis. Int. J. Surg..

[17] Yuan Y., He W., Yang Z., Qiu J., Huang Z., Shi Y., Lin Z., Zheng Y., Chen M., Lau W.Y., Li B., Yuan Y. (2023). TACE-HAIC combined with targeted therapy and immunotherapy versus TACE alone for hepatocellular carcinoma with portal vein tumour thrombus: a propensity score matching study. Int. J. Surg..

[18] Deng M., Cai H., He B., Guan R., Lee C., Guo R. (2023). Hepatic arterial infusion chemotherapy versus transarterial chemoembolization, potential conversion therapies for single huge hepatocellular carcinoma: a retrospective comparison study. Int. J. Surg..

[19] Si T., Shao Q., Jassem W., Ma Y., Heaton N. (2025). Optimal candidates and surrogate endpoints for HAIC versus Sorafenib in hepatocellular carcinoma: an updated systematic review and meta-analysis. Int. J. Surg..

[20] Huang Z., Chen T., Li W., He W., Liu S., Wu Z., Li B., Yuan Y., Qiu J. (2024). Atezolizumab and bevacizumab plus transarterial chemoembolization and hepatic arterial infusion chemotherapy for patients with high tumor burden unresectable hepatocellular carcinoma: a multi-center cohort study. Int. Immunopharmacol..

[21] Regnault H., Chalaye J., Galetto-Pregliasco A., Perrin C., Derbel H., Amaddeo G., Mulé S., Lequoy M., Kobeiter H., Reizine E., Itti E., Duvoux C., Laurent A., Leroy V., Sommacale D., Rasolonirina D., Luciani A., Calderaro J., Tacher V., Brustia R. (2024). Selective internal radiation therapy for unresectable HCC: the SIRT downstaging study. Hepatol Commun.

[22] Liu D.M., Leung T.W., Chow P.K., Ng D.C., Lee R.C., Kim Y.H., Mao Y., Cheng Y.F., Teng G.J., Lau W.Y. (2022). Clinical consensus statement: selective internal radiation therapy with yttrium 90 resin microspheres for hepatocellular carcinoma in Asia. Int. J. Surg..

[23] Edeline J., Touchefeu Y., Guiu B., Farge O., Tougeron D., Baumgaertner I., Ayav A., Campillo-Gimenez B., Beuzit L., Pracht M., Lièvre A., Le Sourd S., Boudjema K., Rolland Y., Boucher E., Garin E. (2020). Radioembolization plus chemotherapy for first-line treatment of locally advanced intrahepatic cholangiocarcinoma: a phase 2 clinical trial. JAMA Oncol..

[24] Puleo L., Agate L., Bargellini I., Boni G., Piaggi P., Traino C., Depalo T., Lorenzoni G., Bianchi F., Volterrani D., Brogioni S., Bottici V., Brunetto M.R., Coco B., Molinaro E., Elisei R. (2022). Yttrium-90 transarterial radioembolization for liver metastases from medullary thyroid cancer. Eur. Thyroid J..

[25] Qin S., Chen M., Cheng A.L., Kaseb A.O., Kudo M., Lee H.C., Yopp A.C., Zhou J., Wang L., Wen X., Heo J., Tak W.Y., Nakamura S., Numata K., Uguen T., Hsiehchen D., Cha E., Hack S.P., Lian Q., Ma N., Spahn J.H., Wang Y., Wu C., Chow P.K.H. (2023). Atezolizumab plus bevacizumab versus active surveillance in patients with resected or ablated high-risk hepatocellular carcinoma (IMbrave050): a randomised, open-label, multicentre, phase 3 trial. Lancet.

[26] Chiang C.L., Chiu K.W.H., Chan K.S.K., Lee F.A.S., Li J.C.B., Wan C.W.S., Dai W.C., Lam T.C., Chen W., Wong N.S.M., Cheung A.L.Y., Lee V.W.Y., Lau V.W.H., El Helali A., Man K., Kong F.M.S., Lo C.M., Chan A.C. (2023). Sequential transarterial chemoembolisation and stereotactic body radiotherapy followed by immunotherapy as conversion therapy for patients with locally advanced, unresectable hepatocellular carcinoma (START-FIT): a single-arm, phase 2 trial. Lancet Gastroenterol. Hepatol..

[27] Lee J.H., Tak W.Y., Lee Y., Heo M.K., Song J.S., Kim H.Y., Park S.Y., Bae S.H., Lee J.H., Heo J., Kim K.H., Bae Y.S., Kim Y.J. (2017). Adjuvant immunotherapy with autologous dendritic cells for hepatocellular carcinoma, randomized phase II study. OncoImmunology.

[28] Thomsen L.C.V., Honoré A., Reisæter L.A.R., Almås B., Børretzen A., Helle S.I., Førde K., Kristoffersen E.K., Kaada S.H., Melve G.K., Haslerud T.M., Biermann M., Bigalke I., Kvalheim G., Azeem W., Olsen J.R., Gabriel B., Knappskog S., Halvorsen O.J., Akslen L.A., Bahn D., Pantel K., Riethdorf S., Ragde H., Gjertsen B.T., Øyan A.M., Kalland K.H., Beisland C. (2023). A phase I prospective, non-randomized trial of autologous dendritic cell-based cryoimmunotherapy in patients with metastatic castration-resistant prostate cancer. Cancer Immunol. Immunother..

[29] Yang Y., Qin Z., Du D., Wu Y., Qiu S., Mu F., Xu K., Chen J. (2019). Safety and short-term efficacy of irreversible electroporation and allogenic natural killer cell immunotherapy combination in the treatment of patients with unresectable primary liver cancer. Cardiovasc. Interv. Radiol..

[30] Guo J., Bao X., Liu F., Guo J., Wu Y., Xiong F., Lu J. (2023). Efficacy of invariant natural killer T cell infusion plus transarterial embolization vs transarterial embolization alone for hepatocellular carcinoma patients: a phase 2 randomized clinical trial. J. Hepatocell. Carcinoma.

[31] Lin M., Zhang X., Liang S., Luo H., Alnaggar M., Liu A., Yin Z., Chen J., Niu L., Jiang Y. (2020). Irreversible electroporation plus allogenic Vγ9Vδ2 T cells enhances antitumor effect for locally advanced pancreatic cancer patients. Signal Transduct Target Ther.

[32] Katz S.C., Hardaway J., Prince E., Guha P., Cunetta M., Moody A., Wang L.J., Armenio V., Espat N.J., Junghans R.P. (2020). HITM-SIR: phase Ib trial of intraarterial chimeric antigen receptor T-cell therapy and selective internal radiation therapy for CEA(+) liver metastases. Cancer Gene Ther..

[33] Brown Z.J., Heinrich B., Greten T.F. (2018). Mouse models of hepatocellular carcinoma: an overview and highlights for immunotherapy research. Nat. Rev. Gastroenterol. Hepatol..

[34] Zhu S., Wu Y., Zhang X., Peng S., Xiao H., Chen S., Xu L., Su T., Kuang M. (2023). Targeting N(7)-methylguanosine tRNA modification blocks hepatocellular carcinoma metastasis after insufficient radiofrequency ablation. Mol. Ther..

[35] Lyu N., Kong Y., Li X., Mu L., Deng H., Chen H., He M., Lai J., Li J., Tang H., Lin Y., Zhao M. (2020). Ablation reboots the response in advanced hepatocellular carcinoma with stable or atypical response during PD-1 therapy: a proof-of-concept study. Front. Oncol..

[36] Pan H., Yu M., Tang X., Mao X., Liu M., Zhang K., Qian C., Wang J., Xie H., Qiu W., Ding Q., Wang S., Zhou W. (2024). Preoperative single-dose camrelizumab and/or microwave ablation in women with early-stage breast cancer: a window-of-opportunity trial. Med.

[37] Duffy A.G., Ulahannan S.V., Makorova-Rusher O., Rahma O., Wedemeyer H., Pratt D., Davis J.L., Hughes M.S., Heller T., ElGindi M., Uppala A., Korangy F., Kleiner D.E., Figg W.D., Venzon D., Steinberg S.M., Venkatesan A.M., Krishnasamy V., Abi-Jaoudeh N., Levy E., Wood B.J., Greten T.F. (2017). Tremelimumab in combination with ablation in patients with advanced hepatocellular carcinoma. J. Hepatol..

[38] Xie C., Duffy A.G., Mabry-Hrones D., Wood B., Levy E., Krishnasamy V., Khan J., Wei J.S., Agdashian D., Tyagi M., Gangalapudi V., Fioravanti S., Walker M., Anderson V., Venzon D., Figg W.D., Sandhu M., Kleiner D.E., Morelli M.P., Floudas C.S., Brar G., Steinberg S.M., Korangy F., Greten T.F. (2019). Tremelimumab in combination with microwave ablation in patients with refractory biliary tract cancer. Hepatology.

[39] Campbell M.T., Matin S.F., Tam A.L., Sheth R.A., Ahrar K., Tidwell R.S., Rao P., Karam J.A., Wood C.G., Tannir N.M., Jonasch E., Gao J., Zurita A.J., Shah A.Y., Jindal S., Duan F., Basu S., Chen H., Espejo A.B., Allison J.P., Yadav S.S., Sharma P. (2021). Pilot study of Tremelimumab with and without cryoablation in patients with metastatic renal cell carcinoma. Nat. Commun..

[40] Monge C., Xie C., Myojin Y., Coffman-D'Annibale K.L., Hrones D., Brar G., Wang S., Budhu A., Figg W.D., Cam M., Finney R., Levy E.B., Kleiner D.E., Steinberg S.M., Wang X.W., Redd B., Wood B.J., Greten T.F. (2024). Combined immune checkpoint inhibition with durvalumab and tremelimumab with and without radiofrequency ablation in patients with advanced biliary tract carcinoma. Cancer Med..

[41] Barqawi A.B., Rodrigues Pessoa R., Crawford E.D., Al-Musawi M., MacDermott T., O'Donell C., Kendl R.M. (2021). Boosting immune response with GM-CSF optimizes primary cryotherapy outcomes in the treatment of prostate cancer: a prospective randomized clinical trial. Prostate Cancer Prostatic Dis..

[42] Bui N.Q., Nemat-Gorgani N., Subramanian A., Torres I.A., Lohman M., Sears T.J., van de Rijn M., Charville G.W., Becker H.C., Wang D.S., Hwang G.L., Ganjoo K.N., Moding E.J. (2023). Monitoring sarcoma response to immune checkpoint inhibition and local cryotherapy with circulating tumor DNA analysis. Clin. Cancer Res..

[43] Yang X., Liao Y., Fan L., Lin B., Li J., Wu D., Liao D., Yuan L., Liu J., Gao F., Feng G., Du X. (2024). High-intensity focused ultrasound ablation combined with immunotherapy for treating liver metastases: a prospective non-randomized trial. PLoS One.

[44] Domingo-Musibay E., Heun J.M., Nevala W.K., Callstrom M., Atwell T., Galanis E., Erickson L.A., Markovic S.N. (2017). Endogenous heat-shock protein induction with or without radiofrequency ablation or cryoablation in patients with stage IV melanoma. Oncologist.

[45] Zhang T., Chen J., Niu L., Liu Y., Ye G., Jiang M., Qi Z. (2022). Clinical safety and efficacy of locoregional therapy combined with adoptive transfer of allogeneic γδ T cells for advanced hepatocellular carcinoma and intrahepatic cholangiocarcinoma. J Vasc Interv Radiol.

[46] Cai J., Zhao J., Liu D., Xie H., Qi H., Ma J., Sun Z., Zhao H. (2021). Efficacy and safety of central memory T cells combined with adjuvant therapy to prevent recurrence of hepatocellular carcinoma with microvascular invasion: a pilot study. Front. Oncol..

[47] Guo C., Zhang J., Huang X., Chen Y., Sheng J., Huang X., Sun J., Xiao W., Sun K., Gao S., Que R., Shen Y., Zhang M., Wu J., Bai X., Liang T. (2023). Preoperative sintilimab plus transarterial chemoembolization for hepatocellular carcinoma exceeding the Milan criteria: a phase II trial. Hepatol Commun.

[48] Pinato D.J., D'Alessio A., Fulgenzi C.A.M., Schlaak A.E., Celsa C., Killmer S., Blanco J.M., Ward C., Stikas C.V., Openshaw M.R., Acuti N., Nteliopoulos G., Balcells C., Keun H.C., Goldin R.D., Ross P.J., Cortellini A., Thomas R., Young A.M., Danckert N., Tait P., Marchesi J.R., Bengsch B., Sharma R. (2024). Safety and preliminary efficacy of pembrolizumab following transarterial chemoembolization for hepatocellular carcinoma: the PETAL phase ib study. Clin. Cancer Res..

[49] Wu X.K., Yang L.F., Chen Y.F., Chen Z.W., Lu H., Shen X.Y., Chi M.H., Wang L., Zhang H., Chen J.F., Huang J.Y., Zeng Y.Y., Yan M.L., Zhang Z.B. (2024). Transcatheter arterial chemoembolisation combined with lenvatinib plus camrelizumab as conversion therapy for unresectable hepatocellular carcinoma: a single-arm, multicentre, prospective study. eClinicalMedicine.

[50] de la Torre-Aláez M., Matilla A., Varela M., Iñarrairaegui M., Reig M., Lledó J.L., Arenas J.I., Lorente S., Testillano M., Márquez L., Da Fonseca L., Argemí J., Gómez-Martin C., Rodriguez-Fraile M., Bilbao J.I., Sangro B. (2022). Nivolumab after selective internal radiation therapy for the treatment of hepatocellular carcinoma: a phase 2, single-arm study. J. Immunother. Cancer.

[51] Musiu C., Adamo A., Caligola S., Agostini A., Frusteri C., Lupo F., Boschi F., Busato A., Poffe O., Anselmi C., Vella A., Wang T., Dusi S., Piro G., Carbone C., Tortora G., Marzola P., D'Onofrio M., Crinò S.F., Corbo V., Scarpa A., Salvia R., Malleo G., Lionetto G., Sartoris S., Ugel S., Bassi C., Bronte V., Paiella S., De Sanctis F. (2025). Local ablation disrupts immune evasion in pancreatic cancer. Cancer Lett..

[52] Karimi A., Yarmohammadi H., Erinjeri J.P. (2024). Immune effects of intra-arterial liver-directed therapies. J Vasc Interv Radiol.

[53] Takaki H., Imai N., Contessa T.T., Srimathveeravalli G., Covey A.M., Getrajdman G.I., Brown K.T., Solomon S.B., Erinjeri J.P. (2016). Peripheral blood regulatory T-cell and type 1 helper T-cell population decrease after hepatic artery embolization. J Vasc Interv Radiol.

[54] Lee H.L., Jang J.W., Lee S.W., Yoo S.H., Kwon J.H., Nam S.W., Bae S.H., Choi J.Y., Han N.I., Yoon S.K. (2019). Inflammatory cytokines and change of Th1/Th2 balance as prognostic indicators for hepatocellular carcinoma in patients treated with transarterial chemoembolization. Sci. Rep..

[55] Wu Y., Fan W., Xue M., Zhong B., Zhang S., Wang Y., Yao W., Zhao Y., Li J. (2019). Postintervention interleukin-6 (IL-6) level, rather than the pretreatment or dynamic changes of IL-6, as an early practical marker of tumor response in hepatocellular carcinoma treated with transarterial chemoembolization. Oncologist.

[56] Chew V., Lee Y.H., Pan L., Nasir N.J.M., Lim C.J., Chua C., Lai L., Hazirah S.N., Lim T.K.H., Goh B.K.P., Chung A., Lo R.H.G., Ng D., Filarca R.L.F., Albani S., Chow P.K.H. (2019). Immune activation underlies a sustained clinical response to Yttrium-90 radioembolisation in hepatocellular carcinoma. Gut.

[57] Deipolyi A.R., Johnson C.B., Riedl C.C., Kunin H., Solomon S.B., Oklu R., Hsu M., Moskowitz C.S., Kombak F.E., Bhanot U., Erinjeri J.P. (2023). Prospective evaluation of immune activation associated with response to radioembolization assessed with PET/CT in women with breast cancer liver metastasis. Radiology.

[58] Tischfield D.J., Gurevich A., Johnson O., Gatmaytan I., Nadolski G.J., Soulen M.C., Kaplan D.E., Furth E., Hunt S.J., Gade T.P.F. (2022). Transarterial embolization modulates the immune response within target and nontarget hepatocellular carcinomas in a rat model. Radiology.

[59] Kudo M. (2024). A changing role of transarterial chemoembolization in the era of immune checkpoint inhibitor plus anti-VEGF/TKI plus transarterial chemoembolization: from total embolization to partial embolization (immune boost transarterial chemoembolization). Liver Cancer.

[60] Kudo M., Aoki T., Ueshima K., Tsuchiya K., Morita M., Chishina H., Takita M., Hagiwara S., Minami Y., Ida H., Nishida N., Ogawa C., Tomonari T., Nakamura N., Kuroda H., Takebe A., Takeyama Y., Hidaka M., Eguchi S., Chan S.L., Kurosaki M., Izumi N. (2023). Achievement of complete response and drug-free status by atezolizumab plus bevacizumab combined with or without curative conversion in patients with transarterial chemoembolization-unsuitable, intermediate-stage hepatocellular carcinoma: a multicenter proof-of-concept study. Liver Cancer.

[61] Kudo M. (2024). Immune checkpoint inhibitors plus anti-VEGF/tyrosine kinase inhibitors combined with TACE (triple therapy) in unresectable hepatocellular carcinoma. Liver Cancer.

[62] Craciun L., de Wind R., Demetter P., Lucidi V., Bohlok A., Michiels S., Bouazza F., Vouche M., Tancredi I., Verset G., Garaud S., Naveaux C., Galdon M.G., Gallo K.W., Hendlisz A., Derijckere I.D., Flamen P., Larsimont D., Donckier V. (2020). Retrospective analysis of the immunogenic effects of intra-arterial locoregional therapies in hepatocellular carcinoma: a rationale for combining selective internal radiation therapy (SIRT) and immunotherapy. BMC Cancer.

[63] Chen Z., Meng L., Zhang J., Zhang X. (2023). Progress in the cryoablation and cryoimmunotherapy for tumor. Front. Immunol..

[64] He F., He Z., Wang C. (2024). A novel role of AIM2 inflammasome-mediated pyroptosis in radiofrequency ablation of hepatocellular carcinoma. Ann. Hepatol..

[65] Yu X., Li X., Chen Q., Wang S., Xu R., He Y., Qin X., Zhang J., Yang W., Shi L., Lu L., Zheng Y., Pang Z., Peng S. (2024). High intensity focused ultrasound-driven nanomotor for effective ferroptosis-immunotherapy of TNBC. Adv. Sci. (Weinh.).

[66] Segawa K., Nagata S. (2015). An apoptotic 'eat me' signal: phosphatidylserine exposure. Trends Cell Biol..

[67] Chang M., Hou Z., Wang M., Li C., Lin J. (2021). Recent advances in hyperthermia therapy-based synergistic immunotherapy. Adv Mater.

[68] Dromi S.A., Walsh M.P., Herby S., Traughber B., Xie J., Sharma K.V., Sekhar K.P., Luk A., Liewehr D.J., Dreher M.R., Fry T.J., Wood B.J. (2009). Radiofrequency ablation induces antigen-presenting cell infiltration and amplification of weak tumor-induced immunity. Radiology.

[69] Mizukoshi E., Yamashita T., Arai K., Sunagozaka H., Ueda T., Arihara F., Kagaya T., Yamashita T., Fushimi K., Kaneko S. (2013). Enhancement of tumor-associated antigen-specific T cell responses by radiofrequency ablation of hepatocellular carcinoma. Hepatology.

[70] Leuchte K., Staib E., Thelen M., Gödel P., Lechner A., Zentis P., Garcia-Marquez M., Waldschmidt D., Datta R.R., Wahba R., Wybranski C., Zander T., Quaas A., Drebber U., Stippel D.L., Bruns C., von Bergwelt-Baildon M., Wennhold K., Schlößer H.A. (2021). Microwave ablation enhances tumor-specific immune response in patients with hepatocellular carcinoma. Cancer Immunol. Immunother..

[71] Nobuoka D., Motomura Y., Shirakawa H., Yoshikawa T., Kuronuma T., Takahashi M., Nakachi K., Ishii H., Furuse J., Gotohda N., Takahashi S., Nakagohri T., Konishi M., Kinoshita T., Komori H., Baba H., Fujiwara T., Nakatsura T. (2012). Radiofrequency ablation for hepatocellular carcinoma induces glypican-3 peptide-specific cytotoxic T lymphocytes. Int. J. Oncol..

[72] Tasu J.P., Tougeron D., Rols M.P. (2022). Irreversible electroporation and electrochemotherapy in oncology: state of the art. Diagn Interv Imaging.

[73] Dai Z., Wang Z., Lei K., Liao J., Peng Z., Lin M., Liang P., Yu J., Peng S., Chen S., Kuang M. (2021). Irreversible electroporation induces CD8(+) T cell immune response against post-ablation hepatocellular carcinoma growth. Cancer Lett..

[74] Burbach B.J., O'Flanagan S.D., Shao Q., Young K.M., Slaughter J.R., Rollins M.R., Street T.J.L., Granger V.E., Beura L.K., Azarin S.M., Ramadhyani S., Forsyth B.R., Bischof J.C., Shimizu Y. (2021). Irreversible electroporation augments checkpoint immunotherapy in prostate cancer and promotes tumor antigen-specific tissue-resident memory CD8+ T cells. Nat. Commun..

[75] Zhao J., Wen X., Tian L., Li T., Xu C., Wen X., Melancon M.P., Gupta S., Shen B., Peng W., Li C. (2019). Irreversible electroporation reverses resistance to immune checkpoint blockade in pancreatic cancer. Nat. Commun..

[76] Qu S., Worlikar T., Felsted A.E., Ganguly A., Beems M.V., Hubbard R., Pepple A.L., Kevelin A.A., Garavaglia H., Dib J., Toma M., Huang H., Tsung A., Xu Z., Cho C.S. (2020). Non-thermal histotripsy tumor ablation promotes abscopal immune responses that enhance cancer immunotherapy. J. Immunother. Cancer.

[77] Thim E.A., Kitelinger L.E., Rivera-Escalera F., Mathew A.S., Elliott M.R., Bullock T.N.J., Price R.J. (2024). Focused ultrasound ablation of melanoma with boiling histotripsy yields abscopal tumor control and antigen-dependent dendritic cell activation. Theranostics.

[78] Pepple A.L., Guy J.L., McGinnis R., Felsted A.E., Song B., Hubbard R., Worlikar T., Garavaglia H., Dib J., Chao H., Boyle N., Olszewski M., Xu Z., Ganguly A., Cho C.S. (2023). Spatiotemporal local and abscopal cell death and immune responses to histotripsy focused ultrasound tumor ablation. Front. Immunol..

[79] Qi T., Jing Y., Deng J., Chang J., Sun W., Yang R., Liu X., Zhang Q., Wan M., Lu M. (2023). Boiling histotripsy using dual-frequency protocol on murine breast tumor model and promotes immune activation. IEEE Trans Ultrason Ferroelectr Freq Control.

[80] Hay A.N., Imran K.M., Hendricks-Wenger A., Gannon J.M., Sereno J., Simon A., Lopez V.A., Coutermarsh-Ott S., Vlaisavljevich E., Allen I.C., Tuohy J.L. (2023). Ablative and immunostimulatory effects of histotripsy ablation in a murine osteosarcoma model. Biomedicines.

[81] Chu K.F., Dupuy D.E. (2014). Thermal ablation of tumours: biological mechanisms and advances in therapy. Nat. Rev. Cancer.

[82] Slovak R., Ludwig J.M., Gettinger S.N., Herbst R.S., Kim H.S. (2017). Immuno-thermal ablations - boosting the anticancer immune response. J. Immunother. Cancer.

[83] Guan Q., Gu J., Zhang H., Ren W., Ji W., Fan Y. (2015). Correlation between vascular endothelial growth factor levels and prognosis of hepatocellular carcinoma patients receiving radiofrequency ablation. Biotechnol. Biotechnol. Equip..

[84] Finn R.S., Qin S., Ikeda M., Galle P.R., Ducreux M., Kim T.Y., Kudo M., Breder V., Merle P., Kaseb A.O., Li D., Verret W., Xu D.Z., Hernandez S., Liu J., Huang C., Mulla S., Wang Y., Lim H.Y., Zhu A.X., Cheng A.L. (2020). Atezolizumab plus bevacizumab in unresectable hepatocellular carcinoma. N. Engl. J. Med..

[85] Wei X., Chen Y., Jiang X., Peng M., Liu Y., Mo Y., Ren D., Hua Y., Yu B., Zhou Y., Liao Q., Wang H., Xiang B., Zhou M., Li X., Li G., Li Y., Xiong W., Zeng Z. (2021). Mechanisms of vasculogenic mimicry in hypoxic tumor microenvironments. Mol. Cancer.

[86] Joshi S., Singh A.R., Zulcic M., Durden D.L. (2014). A macrophage-dominant PI3K isoform controls hypoxia-induced HIF1α and HIF2α stability and tumor growth, angiogenesis, and metastasis. Mol. Cancer Res..

[87] Chen K., Wang Q., Liu X., Wang F., Yang Y., Tian X. (2022). Hypoxic pancreatic cancer derived exosomal miR-30b-5p promotes tumor angiogenesis by inhibiting GJA1 expression. Int. J. Biol. Sci..

[88] Yang M., Mu Y., Yu X., Gao D., Zhang W., Li Y., Liu J., Sun C., Zhuang J. (2024). Survival strategies: how tumor hypoxia microenvironment orchestrates angiogenesis. Biomed. Pharmacother..

[89] Fang C., Dai L., Wang C., Fan C., Yu Y., Yang L., Deng H., Zhou Z. (2021). Secretogranin II impairs tumor growth and angiogenesis by promoting degradation of hypoxia-inducible factor-1α in colorectal cancer. Mol. Oncol..

[90] Vignali P.D.A., DePeaux K., Watson M.J., Ye C., Ford B.R., Lontos K., McGaa N.K., Scharping N.E., Menk A.V., Robson S.C., Poholek A.C., Rivadeneira D.B., Delgoffe G.M. (2023). Hypoxia drives CD39-dependent suppressor function in exhausted T cells to limit antitumor immunity. Nat. Immunol..

[91] Moussa M., Goldberg S.N., Kumar G., Sawant R.R., Levchenko T., Torchilin V., Ahmed M. (2014). Radiofrequency ablation-induced upregulation of hypoxia-inducible factor-1α can be suppressed with adjuvant bortezomib or liposomal chemotherapy. J Vasc Interv Radiol.

[92] Xiao E.H., Guo D., Bian D.J. (2009). Effect of preoperative transcatheter arterial chemoembolization on angiogenesis of hepatocellular carcinoma cells. World J. Gastroenterol..

[93] Gadaleta C.D., Ranieri G. (2011). Trans-arterial chemoembolization as a therapy for liver tumours: new clinical developments and suggestions for combination with angiogenesis inhibitors. Crit. Rev. Oncol. Hematol..

[94] Pleguezuelo M., Marelli L., Misseri M., Germani G., Calvaruso V., Xiruochakis E., Manousou P., Burroughs A.K. (2008). TACE versus TAE as therapy for hepatocellular carcinoma. Expert Rev. Anticancer Ther..

[95] Savic L.J., Chen E., Nezami N., Murali N., Hamm C.A., Wang C., Lin M., Schlachter T., Hong K., Georgiades C., Chapiro J., Laage Gaupp F.M. (2022). Conventional vs. Drug-eluting beads transarterial chemoembolization for unresectable hepatocellular carcinoma-A propensity score weighted comparison of efficacy and safety. Cancers (Basel).

[96] Sergio A., Cristofori C., Cardin R., Pivetta G., Ragazzi R., Baldan A., Girardi L., Cillo U., Burra P., Giacomin A., Farinati F. (2008). Transcatheter arterial chemoembolization (TACE) in hepatocellular carcinoma (HCC): the role of angiogenesis and invasiveness. Am. J. Gastroenterol..

[97] Sen A., Capitano M.L., Spernyak J.A., Schueckler J.T., Thomas S., Singh A.K., Evans S.S., Hylander B.L., Repasky E.A. (2011). Mild elevation of body temperature reduces tumor interstitial fluid pressure and hypoxia and enhances efficacy of radiotherapy in murine tumor models. Cancer Res..

[98] Lerner E.C., Edwards R.M., Wilkinson D.S., Fecci P.E. (2022). Laser ablation: heating up the anti-tumor response in the intracranial compartment. Adv. Drug Deliv. Rev..

[99] Pan J., Xu Y., Wu Q., Hu P., Shi J. (2021). Mild magnetic hyperthermia-activated innate immunity for liver cancer therapy. J. Am. Chem. Soc..

[100] Chen Q., Fisher D.T., Clancy K.A., Gauguet J.M., Wang W.C., Unger E., Rose-John S., von Andrian U.H., Baumann H., Evans S.S. (2006). Fever-range thermal stress promotes lymphocyte trafficking across high endothelial venules via an interleukin 6 trans-signaling mechanism. Nat. Immunol..

[101] Shen X., Chen T., Liu N., Yang B., Feng G., Yu P., Zhan C., Yin N., Wang Y., Huang B., Chen S. (2022). MRI-guided microwave ablation and albumin-bound paclitaxel for lung tumors: initial experience. Front. Bioeng. Biotechnol..

[102] Zhao S., Peralta R.M., Avina-Ochoa N., Delgoffe G.M., Kaech S.M. (2021). Metabolic regulation of T cells in the tumor microenvironment by nutrient availability and diet. Semin. Immunol..

[103] Peralta R.M., Xie B., Lontos K., Nieves-Rosado H., Spahr K., Joshi S., Ford B.R., Quann K., Frisch A.T., Dean V., Philbin M., Cillo A.R., Gingras S., Poholek A.C., Kane L.P., Rivadeneira D.B., Delgoffe G.M. (2024). Dysfunction of exhausted T cells is enforced by MCT11-mediated lactate metabolism. Nat. Immunol..

[104] Kovacs Z.I., Kim S., Jikaria N., Qureshi F., Milo B., Lewis B.K., Bresler M., Burks S.R., Frank J.A. (2017). Disrupting the blood-brain barrier by focused ultrasound induces sterile inflammation. Proc. Natl. Acad. Sci. U. S. A..

[105] Jung O., Thomas A., Burks S.R., Dustin M.L., Frank J.A., Ferrer M., Stride E. (2022). Neuroinflammation associated with ultrasound-mediated permeabilization of the blood-brain barrier. Trends Neurosci..

[106] Choi H.J., Han M., Seo H., Park C.Y., Lee E.H., Park J. (2022). The new insight into the inflammatory response following focused ultrasound-mediated blood-brain barrier disruption. Fluids Barriers CNS.

[107] Lian X., Yang K., Li R., Li M., Zuo J., Zheng B., Wang W., Wang P., Zhou S. (2022). Immunometabolic rewiring in tumorigenesis and anti-tumor immunotherapy. Mol. Cancer.

[108] Wang K., Zerdes I., Johansson H.J., Sarhan D., Sun Y., Kanellis D.C., Sifakis E.G., Mezheyeuski A., Liu X., Loman N., Hedenfalk I., Bergh J., Bartek J., Hatschek T., Lehtiö J., Matikas A., Foukakis T. (2024). Longitudinal molecular profiling elucidates immunometabolism dynamics in breast cancer. Nat. Commun..

[109] Huang C.X., Lao X.M., Wang X.Y., Ren Y.Z., Lu Y.T., Shi W., Wang Y.Z., Wu C.Y., Xu L., Chen M.S., Gao Q., Liu L., Wei Y., Kuang D.M. (2024). Pericancerous cross-presentation to cytotoxic T lymphocytes impairs immunotherapeutic efficacy in hepatocellular carcinoma. Cancer Cell.

[110] Sahai E., Astsaturov I., Cukierman E., DeNardo D.G., Egeblad M., Evans R.M., Fearon D., Greten F.R., Hingorani S.R., Hunter T., Hynes R.O., Jain R.K., Janowitz T., Jorgensen C., Kimmelman A.C., Kolonin M.G., Maki R.G., Powers R.S., Puré E., Ramirez D.C., Scherz-Shouval R., Sherman M.H., Stewart S., Tlsty T.D., Tuveson D.A., Watt F.M., Weaver V., Weeraratna A.T., Werb Z. (2020). A framework for advancing our understanding of cancer-associated fibroblasts. Nat. Rev. Cancer.

[111] Koikawa K., Kibe S., Suizu F., Sekino N., Kim N., Manz T.D., Pinch B.J., Akshinthala D., Verma A., Gaglia G., Nezu Y., Ke S., Qiu C., Ohuchida K., Oda Y., Lee T.H., Wegiel B., Clohessy J.G., London N., Santagata S., Wulf G.M., Hidalgo M., Muthuswamy S.K., Nakamura M., Gray N.S., Zhou X.Z., Lu K.P. (2021). Targeting Pin1 renders pancreatic cancer eradicable by synergizing with immunochemotherapy. Cell.

[112] Koda M., Maeda Y., Matsunaga Y., Mimura K., Murawaki Y., Horie Y. (2003). Hepatocellular carcinoma with sarcomatous change arising after radiofrequency ablation for well-differentiated hepatocellular carcinoma. Hepatol. Res..

[113] Kang T.W., Lim H.K., Cha D.I. (2017). Aggressive tumor recurrence after radiofrequency ablation for hepatocellular carcinoma. Clin. Mol. Hepatol..

[114] Nijkamp M.W., Hoogwater F.J., Steller E.J., Westendorp B.F., van der Meulen T.A., Leenders M.W., Borel Rinkes I.H., Kranenburg O. (2010). CD95 is a key mediator of invasion and accelerated outgrowth of mouse colorectal liver metastases following radiofrequency ablation. J. Hepatol..

[115] Kang T.W., Lim H.K., Lee M.W., Kim Y.S., Rhim H., Lee W.J., Gwak G.Y., Paik Y.H., Lim H.Y., Kim M.J. (2015). Aggressive intrasegmental recurrence of hepatocellular carcinoma after radiofrequency ablation: risk factors and clinical significance. Radiology.

[116] Zhang G.P., Xie Z.L., Jiang J., Zhao Y.T., Lei K., Lin Z.L., Chen S.L., Su T.H., Tan L., Peng S., Wang J., Liu C., Kuang M. (2023). Mechanical confinement promotes heat resistance of hepatocellular carcinoma via SP1/IL4I1/AHR axis. Cell Rep. Med..

[117] Su T., Huang M., Liao J., Lin S., Yu P., Yang J., Cai Y., Zhu S., Xu L., Peng Z., Peng S., Chen S., Kuang M. (2021). Insufficient radiofrequency ablation promotes hepatocellular carcinoma metastasis through N6-methyladenosine mRNA methylation-dependent mechanism. Hepatology.

[118] Zeng X., Liao G., Li S., Liu H., Zhao X., Li S., Lei K., Zhu S., Chen Z., Zhao Y., Ren X., Su T., Cheng A.S., Peng S., Lin S., Wang J., Chen S., Kuang M. (2023). Eliminating METTL1-mediated accumulation of PMN-MDSCs prevents hepatocellular carcinoma recurrence after radiofrequency ablation. Hepatology.

[119] Zhou J., Wan J., Gao X., Zhang X., Jaffrey S.R., Qian S.B. (2015). Dynamic m(6)A mRNA methylation directs translational control of heat shock response. Nature.

[120] Wilkinson E., Cui Y.H., He Y.Y. (2021). Context-dependent roles of RNA modifications in stress responses and diseases. Int. J. Mol. Sci..

[121] Shi L., Wang J., Ding N., Zhang Y., Zhu Y., Dong S., Wang X., Peng C., Zhou C., Zhou L., Li X., Shi H., Wu W., Long X., Wu C., Liao W. (2019). Inflammation induced by incomplete radiofrequency ablation accelerates tumor progression and hinders PD-1 immunotherapy. Nat. Commun..

[122] Tan J., Fan W., Liu T., Zhu B., Liu Y., Wang S., Wu J., Liu J., Zou F., Wei J., Liu L., Zhang X., Zhuang J., Wang Y., Lin H., Huang X., Chen S., Kuang M., Li J. (2023). TREM2(+) macrophages suppress CD8(+) T-cell infiltration after transarterial chemoembolisation in hepatocellular carcinoma. J. Hepatol..

[123] Ueshima E., Nishiofuku H., Takaki H., Hirata Y., Kodama H., Tanaka T., Kichikawa K., Yamakado K., Okada T., Sofue K., Yamaguchi M., Sugimoto K., Murakami T. (2020). Hepatic artery embolization induces the local overexpression of transforming growth factor β1 in a rat hepatoma model. Liver Cancer.

[124] Haen S.P., Gouttefangeas C., Schmidt D., Boss A., Clasen S., von Herbay A., Kosan B., Aebert H., Pereira P.L., Rammensee H.G. (2011). Elevated serum levels of heat shock protein 70 can be detected after radiofrequency ablation. Cell Stress Chaperones.

[125] Ahmed M., Kumar G., Gourevitch S., Levchenko T., Galun E., Torchilin V., Goldberg S.N. (2018). Radiofrequency ablation (RFA)-induced systemic tumor growth can be reduced by suppression of resultant heat shock proteins. Int J Hyperthermia.

[126] Yu M., Pan H., Che N., Li L., Wang C., Wang Y., Ma G., Qian M., Liu J., Zheng M., Xie H., Ling L., Zhao Y., Guan X., Ding Q., Zhou W., Wang S. (2021). Microwave ablation of primary breast cancer inhibits metastatic progression in model mice via activation of natural killer cells. Cell. Mol. Immunol..

[127] Zerbini A., Pilli M., Laccabue D., Pelosi G., Molinari A., Negri E., Cerioni S., Fagnoni F., Soliani P., Ferrari C., Missale G. (2010). Radiofrequency thermal ablation for hepatocellular carcinoma stimulates autologous NK-cell response. Gastroenterology.

[128] Zhou W., Yu M., Mao X., Pan H., Tang X., Wang J., Che N., Xie H., Ling L., Zhao Y., Liu X., Wang C., Zhang K., Qiu W., Ding Q., Wang S. (2022). Landscape of the peripheral immune response induced by local microwave ablation in patients with breast cancer. Adv. Sci. (Weinh.).

[129] Ko K.W.S., Chiang J.B., Poon W.L., Lai E., Garnon J. (2021). Abscopal effect after MRI-guided cryoablation of multifocal chest wall desmoid-type fibromatosis. Cardiovasc. Interv. Radiol..

[130] Kumar A.V., Patterson S.G., Plaza M.J. (2019). Abscopal effect following cryoablation of breast cancer. J Vasc Interv Radiol.

[131] Shen L., Tan H., Nie J., Jiang Y., Nuerhashi G., Qi H., Cao F., Wen C., Chen S., Zhang T., Zheng W., Liu P., Liu Y., Huang T., Li D., Zhang X., Fan W. (2024). Size selection of intrahepatic lesions for cryoablation contributes to abscopal effect and long-term survival in patients with liver metastatic melanoma receiving PD-1 blockade therapy. Cancer Immunol. Immunother..

[132] Gu C., Wang X., Wang K., Xie F., Chen L., Ji H., Sun J. (2024). Cryoablation triggers type I interferon-dependent antitumor immunity and potentiates immunotherapy efficacy in lung cancer. J. Immunother. Cancer.

[133] Alshebremi M., Tomchuck S.L., Myers J.T., Kingsley D.T., Eid S., Abiff M., Bonner M., Saab S.T., Choi S.H., Huang A.Y. (2023). Functional tumor cell-intrinsic STING, not host STING, drives local and systemic antitumor immunity and therapy efficacy following cryoablation. J. Immunother. Cancer.

[134] Yang X., Gao X., Xu C., Ni T., Sheng Y., Wang J., Sun X., Yuan J., Zhang L., Wang Y. (2024). Cryoablation synergizes with anti-PD-1 immunotherapy induces an effective abscopal effect in murine model of cervical cancer. Transl. Oncol..

[135] Vidal-Jove J., Serres-Creixams X., Ziemlewicz T.J., Cannata J.M. (2021). Liver histotripsy mediated abscopal effect-case report. IEEE Trans Ultrason Ferroelectr Freq Control.

[136] Shi Q., Zhang W., Zhou Y., Huang S., Yu J., Yang M., Zhang Z., Ma J., Luo J., Rao S., Lu D., Peng S., Cao Y., Liu L., Yan Z. (2024). Hypoxia-activated cascade nanovaccine for synergistic chemoembolization-immune therapy of hepatocellular carcinoma. Biomaterials.

[137] Schmidt S., Bonilla W.V., Reiter A., Stemeseder F., Kleissner T., Oeler D., Berka U., El-Gazzar A., Kiefmann B., Schulha S.C., Raguz J., Habbeddine M., Scheinost M., Qing X., Lauterbach H., Matushansky I., Pinschewer D.D., Orlinger K.K. (2020). Live-attenuated lymphocytic choriomeningitis virus-based vaccines for active immunotherapy of HPV16-positive cancer. OncoImmunology.

[138] Li T., Qian C., Gu Y., Zhang J., Li S., Xia N. (2023). Current progress in the development of prophylactic and therapeutic vaccines. Sci. China Life Sci..

[139] Sayour E.J., Boczkowski D., Mitchell D.A., Nair S.K. (2024). Cancer mRNA vaccines: clinical advances and future opportunities. Nat. Rev. Clin. Oncol..

[140] Kon E., Ad-El N., Hazan-Halevy I., Stotsky-Oterin L., Peer D. (2023). Targeting cancer with mRNA-lipid nanoparticles: key considerations and future prospects. Nat. Rev. Clin. Oncol..

[141] Chen J., Ye Z., Huang C., Qiu M., Song D., Li Y., Xu Q. (2022). Lipid nanoparticle-mediated lymph node-targeting delivery of mRNA cancer vaccine elicits robust CD8(+) T cell response. Proc. Natl. Acad. Sci. U. S. A..

[142] Hazlewood J.E., Dumenil T., Le T.T., Slonchak A., Kazakoff S.H., Patch A.M., Gray L.A., Howley P.M., Liu L., Hayball J.D., Yan K., Rawle D.J., Prow N.A., Suhrbier A. (2021). Injection site vaccinology of a recombinant vaccinia-based vector reveals diverse innate immune signatures. PLoS Pathog..

[143] Guo Z.S., Lu B., Guo Z., Giehl E., Feist M., Dai E., Liu W., Storkus W.J., He Y., Liu Z., Bartlett D.L. (2019). Vaccinia virus-mediated cancer immunotherapy: cancer vaccines and oncolytics. J. Immunother. Cancer.

[144] Li M., Jiang A., Han H., Chen M., Wang B., Cheng Y., Zhang H., Wang X., Dai W., Yang W., Zhang Q., He B. (2024). A trinity nano-vaccine system with spatiotemporal immune effect for the adjuvant cancer therapy after radiofrequency ablation. ACS Nano.

[145] Tian Z., Hu Q., Sun Z., Wang N., He H., Tang Z., Chen W. (2023). A booster for radiofrequency ablation: advanced adjuvant therapy via in situ nanovaccine synergized with anti-programmed death ligand 1 immunotherapy for systemically constraining hepatocellular carcinoma. ACS Nano.

[146] Ghani M.A., Bangar A., Yang Y., Jung E., Sauceda C., Mandt T., Shukla S., Webster N.J.G., Steinmetz N.F., Newton I.G. (2023). Treatment of hepatocellular carcinoma by multimodal in situ vaccination using cryoablation and a plant virus immunostimulant. J Vasc Interv Radiol.

[147] Yu Z., Wang D., Qi Y., Liu J., Zhou T., Rao W., Hu K. (2023). Autologous-cancer-cryoablation-mediated nanovaccine augments systematic immunotherapy. Mater. Horiz..

[148] Han J.H., Lee Y.Y., Shin H.E., Han J., Kang J.M., James Wang C.P., Park J.H., Kim S.N., Yoon J.H., Kwon H.K., Park D.H., Park T.E., Choy Y.B., Kim D.H., Kim T.H., Min J., Kim I.H., Park C.G., Han D.K., Park W. (2022). Image-guided in situ cancer vaccination with combination of multi-functional nano-adjuvant and an irreversible electroporation technique. Biomaterials.

[149] Zhou Q., Gong N., Zhang D., Li J., Han X., Dou J., Huang J., Zhu K., Liang P., Liang X.J., Yu J. (2021). Mannose-derived carbon dots amplify microwave ablation-induced antitumor immune responses by capturing and transferring "danger signals" to dendritic cells. ACS Nano.

[150] Chen M., Tan Y., Hu J., Jiang Y., Wang Z., Liu Z., Chen Q. (2021). Injectable immunotherapeutic thermogel for enhanced immunotherapy post tumor radiofrequency ablation. Small.

[151] Liu X., Zhuang Y., Huang W., Wu Z., Chen Y., Shan Q., Zhang Y., Wu Z., Ding X., Qiu Z., Cui W., Wang Z. (2023). Interventional hydrogel microsphere vaccine as an immune amplifier for activated antitumour immunity after ablation therapy. Nat. Commun..

[152] Han X., Chen J., Chu J., Liang C., Ma Q., Fan Q., Liu Z., Wang C. (2019). Platelets as platforms for inhibition of tumor recurrence post-physical therapy by delivery of anti-PD-L1 checkpoint antibody. J Control Release.

[153] Li X., Liu Y., Ke J., Wang Z., Han M., Wang N., Miao Q., Shao B., Zhou D., Yan F., Ji B. (2024). Enhancing radiofrequency ablation for hepatocellular carcinoma: nano-epidrug effects on immune modulation and antigenicity restoration. Adv Mater.

[154] Peng W., Cao Y., Zhang Y., Zhong A., Zhang C., Wei Z., Liu X., Dong S., Wu J., Xue Y., Wu M., Yao C. (2024). Optimal irreversible electroporation combined with nano-enabled immunomodulatory to boost systemic antitumor immunity. Adv Healthc Mater.

[155] Wang J., Huang C.H., Echeagaray O.H., Amirfakhri S., Blair S.L., Trogler W.C., Kummel A.C., Chen C.C. (2019). Microshell enhanced acoustic adjuvants for immunotherapy in glioblastoma. Adv. Therapeut..

[156] Yang Z., Zhu Y., Dong Z., Li W., Yang N., Wang X., Feng L., Liu Z. (2021). Tumor-killing nanoreactors fueled by tumor debris can enhance radiofrequency ablation therapy and boost antitumor immune responses. Nat. Commun..

[157] Pan J., Hu P., Guo Y., Hao J., Ni D., Xu Y., Bao Q., Yao H., Wei C., Wu Q., Shi J. (2020). Combined magnetic hyperthermia and immune therapy for primary and metastatic tumor treatments. ACS Nano.

[158] Lee S., Ko M.J., Avritscher R., Lewandowski R.J., Kim D.H. (2024). Cryo-nanocatalyst enhances therapeutic efficacy of cryo-immunotherapy through necroptosis and local delivery of programmed death-ligand 1 inhibitors. ACS Nano.

[159] Wang Z., Zhang F., Shao D., Chang Z., Wang L., Hu H., Zheng X., Li X., Chen F., Tu Z., Li M., Sun W., Chen L., Dong W.F. (2019). Janus nanobullets combine photodynamic therapy and magnetic hyperthermia to potentiate synergetic anti-metastatic immunotherapy. Adv. Sci. (Weinh.).

[160] Han X., Wang R., Xu J., Chen Q., Liang C., Chen J., Zhao J., Chu J., Fan Q., Archibong E., Jiang L., Wang C., Liu Z. (2019). In situ thermal ablation of tumors in combination with nano-adjuvant and immune checkpoint blockade to inhibit cancer metastasis and recurrence. Biomaterials.

[161] Ou W., Stewart S., White A., Kwizera E.A., Xu J., Fang Y., Shamul J.G., Xie C., Nurudeen S., Tirada N.P., Lu X., Tkaczuk K.H.R., He X. (2023). In-situ cryo-immune engineering of tumor microenvironment with cold-responsive nanotechnology for cancer immunotherapy. Nat. Commun..

[162] Zhu X., Li T., Wang Q., Yan K., Ma S., Lin Y., Zeng G., Liu J., Cao J., Wang D. (2024). Dual-synergistic nanomodulator alleviates exosomal PD-L1 expression enabling exhausted cytotoxic T lymphocytes rejuvenation for potentiated iRFA-treated hepatocellular carcinoma immunotherapy. ACS Nano.

[163] Qi F., Bao Q., Hu P., Guo Y., Yan Y., Yao X., Shi J. (2024). Mild magnetic hyperthermia-activated immuno-responses for primary bladder cancer therapy. Biomaterials.

[164] Carter T.J., Agliardi G., Lin F.Y., Ellis M., Jones C., Robson M., Richard-Londt A., Southern P., Lythgoe M., Zaw Thin M., Ryzhov V., de Rosales R.T.M., Gruettner C., Abdollah M.R.A., Pedley R.B., Pankhurst Q.A., Kalber T.L., Brandner S., Quezada S., Mulholland P., Shevtsov M., Chester K. (2021). Potential of magnetic hyperthermia to stimulate localized immune activation. Small.

[165] Hu J., Albadawi H., Zhang Z., Salomao M.A., Gunduz S., Rehman S., D'Amone L., Mayer J.L., Omenetto F., Oklu R. (2022). Silk embolic material for catheter-directed endovascular drug delivery. Adv Mater.

[166] Chen X., Wang S., Chen Y., Xin H., Zhang S., Wu D., Xue Y., Zha M., Li H., Li K., Gu Z., Wei W., Ping Y. (2023). Non-invasive activation of intratumoural gene editing for improved adoptive T-cell therapy in solid tumours. Nat. Nanotechnol..

[167] Wu Y., Liu Y., Huang Z., Wang X., Jin Z., Li J., Limsakul P., Zhu L., Allen M., Pan Y., Bussell R., Jacobson A., Liu T., Chien S., Wang Y. (2021). Control of the activity of CAR-T cells within tumours via focused ultrasound. Nat. Biomed. Eng..

[168] Li M., Xie D., Tang X., Yang C., Shen Y., Zhou H., Deng W., Liu J., Cai S., Bai L., Wang Y. (2021). Phototherapy facilitates tumor recruitment and activation of natural killer T cells for potent cancer immunotherapy. Nano Lett..

[169] Kim D., Jo S., Lee D., Kim S.M., Seok J.M., Yeo S.J., Lee J.H., Lee J.J., Lee K., Kim T.D., Park S.A. (2023). NK cells encapsulated in micro/macropore-forming hydrogels via 3D bioprinting for tumor immunotherapy. Biomater. Res..

[170] Li Y., Chen W., Kang Y., Zhen X., Zhou Z., Liu C., Chen S., Huang X., Liu H.J., Koo S., Kong N., Ji X., Xie T., Tao W. (2023). Nanosensitizer-mediated augmentation of sonodynamic therapy efficacy and antitumor immunity. Nat. Commun..

[171] Bae W.K., Lee B.C., Kim H.J., Lee J.J., Chung I.J., Cho S.B., Koh Y.S. (2022). A phase I study of locoregional high-dose autologous natural killer cell therapy with hepatic arterial infusion chemotherapy in patients with locally advanced hepatocellular carcinoma. Front. Immunol..

[172] Sun H., Xing C., Jiang S., Yu K., Dai S., Kong H., Jin Y., Shan Y., Yang W., Wang Z., Xiao J., Wang H., Wang W., Li Z., Shi K. (2022). Long term complete response of advanced hepatocellular carcinoma to glypican-3 specific chimeric antigen receptor T-Cells plus sorafenib, a case report. Front. Immunol..

[173] Wang D., Zhang M., Qiu G., Rong C., Zhu X., Qin G., Kong C., Zhou J., Liang X., Bu Z., Liu J., Luo T., Yang J., Zhang K. (2023). Extracellular matrix viscosity reprogramming by in situ Au bioreactor-boosted microwavegenetics disables tumor escape in CAR-T immunotherapy. ACS Nano.

[174] Tang Y., Shu Z., Zhu M., Li S., Ling Y., Fu Y., Hu Z., Wang J., Yang Z., Liao J., Xu L., Yu M., Peng Z. (2023). Size-tunable nanoregulator-based radiofrequency ablation suppresses MDSCs and their compensatory immune evasion in hepatocellular carcinoma. Adv Healthc Mater.

[175] Shen Y., Chen L., Guan X., Han X., Bo X., Li S., Sun L., Chen Y., Yue W., Xu H. (2021). Tailoring chemoimmunostimulant bioscaffolds for inhibiting tumor growth and metastasis after incomplete microwave ablation. ACS Nano.

[176] Li S., Xu F., Ren X., Tan L., Fu C., Wu Q., Chen Z., Ren J., Huang Z., Meng X. (2023). H(2)S-Reactivating antitumor immune response after microwave thermal therapy for long-term tumor suppression. ACS Nano.

[177] Han J.H., Shin H.E., Lee J., Kang J.M., Park J.H., Park C.G., Han D.K., Kim I.H., Park W. (2022). Combination of metal-phenolic network-based immunoactive nanoparticles and bipolar irreversible electroporation for effective cancer immunotherapy. Small.

[178] Yu B., Zhang W., Kwak K., Choi H., Kim D.H. (2020). Electric pulse responsive magnetic nanoclusters loaded with indoleamine 2,3-dioxygenase inhibitor for synergistic immuno-ablation cancer therapy. ACS Appl. Mater. Interfaces.

[179] Li J., Luo C., Sun T., Zhou Y., Huang X., Wu D., Luo X., Zeng C., Li H. (2024). Hypoxia-specific metal-organic frameworks augment cancer immunotherapy of high-intensity focused ultrasound. ACS Nano.

[180] Zhu Y., Yang Z., Pan Z., Hao Y., Wang C., Dong Z., Li Q., Han Y., Tian L., Feng L., Liu Z. (2022). Metallo-alginate hydrogel can potentiate microwave tumor ablation for synergistic cancer treatment. Sci. Adv..

[181] Yang Y., Wang Y., Zeng F., Chen Y., Chen Z., Yan F. (2024). Ultrasound-visible engineered bacteria for tumor chemo-immunotherapy. Cell Rep. Med..

[182] Singh A., Chandrasekar S.V., Valappil V.T., Scaria J., Ranjan A. (2025). Tumor immunomodulation by nanoparticle and focused ultrasound alters gut microbiome in a sexually dimorphic manner. Theranostics.

[183] Son S., Kim N., You D.G., Yoon H.Y., Yhee J.Y., Kim K., Kwon I.C., Kim S.H. (2017). Antitumor therapeutic application of self-assembled RNAi-AuNP nanoconstructs: combination of VEGF-RNAi and photothermal ablation. Theranostics.

[184] Meng L., Cheng Y., Tong X., Gan S., Ding Y., Zhang Y., Wang C., Xu L., Zhu Y., Wu J., Hu Y., Yuan A. (2018). Tumor oxygenation and hypoxia inducible factor-1 functional inhibition via a reactive oxygen species responsive nanoplatform for enhancing radiation therapy and abscopal effects. ACS Nano.

[185] Hong H., Park C.H., Lee J.S., Kim K., Nath S., Oh M.S., Kim S., Lee C.H., Kim K.H., Choi W.H., Choi K.Y., Park H.S., Lee O.J., Hong I.S., Kim S.H. (2025). Ex vivo enhancement of CD8+ T cell activity using functionalized hydrogel encapsulating tonsil-derived lymphatic endothelial cells. Theranostics.

[186] Huang Y., Li X., Cao J., Wei X., Li Y., Wang Z., Cai X., Li R., Chen J. (2022). Use of dissociation degree in lysosomes to predict metal oxide nanoparticle toxicity in immune cells: machine learning boosts nano-safety assessment. Environ. Int..

